# Plasmonic and Dielectric Metasurfaces for Enhanced Spectroscopic Techniques

**DOI:** 10.3390/bios15070401

**Published:** 2025-06-20

**Authors:** Borja García García, María Gabriela Fernández-Manteca, Dimitrios C. Zografopoulos, Celia Gómez-Galdós, Alain A. Ocampo-Sosa, Luis Rodríguez-Cobo, José Francisco Algorri, Adolfo Cobo

**Affiliations:** 1 Photonics Engineering Group, Universidad de Cantabria, 39005 Santander, Spain; borja.garcia@unican.es (B.G.G.); mariela.fernandez@unican.es (M.G.F.-M.); celia.gomez@unican.es (C.G.-G.); luis.rodriguez@unican.es (L.R.-C.); adolfo.cobo@unican.es (A.C.); 2 Instituto de Investigación Sanitaria Valdecilla, 39011 Santander, Spain; alain.ocampo@scsalud.es; 3 School of Electrical and Computer Engineering, Aristotle University of Thessaloniki, GR-54124 Thessaloniki, Greece; dzogra@ece.auth.gr; 4 Consiglio Nazionale delle Ricerche, Istituto per la Microelettronica e Microsistemi, Via del Fosso del Cavaliere 100, 00133 Roma, Italy; 5 Servicio de Microbiología, Hospital Universitario Marqués de Valdecilla, 39008 Santander, Spain; 6 Centro de Investigación Biomédica en Red de Enfermedades Infecciosas (CIBERINFEC), Instituto de Salud Carlos III, 28029 Madrid, Spain; 7 Centro de Investigación Biomédica en Red en el área temática de Bioingeniería, Biomateriales y Nanomedicina (CIBER-BBN), Instituto de Salud Carlos III, 28029 Madrid, Spain

**Keywords:** spectroscopy, SERS, SEIRA, SEF, metasurfaces

## Abstract

Spectroscopic techniques such as Surface-Enhanced Raman Scattering (SERS), Surface-Enhanced Infrared Absorption (SEIRA), and Surface-Enhanced Fluorescence (SEF) are essential analytical techniques used to study the composition of materials by analyzing the way materials scatter light, absorb infrared radiation or emit fluorescence signals. This provides information about their molecular structure and properties. However, traditional SERS, SEIRA, and SEF techniques can be limited in sensitivity, resolution, and reproducibility, hindering their ability to detect and analyze trace amounts of substances or complex molecular structures. Metasurfaces, a class of engineered two-dimensional metamaterials with unique optical properties, have emerged as a promising tool to overcome these limitations and enhance spectroscopic techniques. This article provides a state-of-the-art overview of metasurfaces for enhanced SERS, SEIRA and SEF, covering their theoretical background, different types, advantages, disadvantages, and potential applications.

## 1. Introduction

Metasurfaces have attracted significant attention in recent years because of their ability to manipulate electromagnetic waves in ways not achievable with natural materials [1,2,3]. They are artificial materials composed of subwavelength-sized building blocks, known as meta-atoms, arranged in a two-dimensional pattern [4]. These scatterers are designed to achieve the desired electromagnetic properties through precise control over the amplitude, phase, and polarization of impinging waves [5].

Historically, the concept of artificially engineered material properties can be traced back to early works, including Wood’s anomalies [6] and Levi-Civita’s boundary relations for vanishingly thin sheets [7], which anticipated the interplay between surface plasmons (SPs) [8] and engineered interfaces. SPs are coherent, localized electron oscillations that form at the interface between two materials with oppositely signed dielectric functions, such as a metal and a dielectric [9]. These excitations carry lower energy than bulk (or volume) plasmons, which instead quantise longitudinal electron motion within the bulk electron gas [10]. By virtue of being confined to an interface, SPs generate exponentially decaying electromagnetic fields both inside and outside the metal. The complete excitation, encompassing the electron oscillations and their accompanying electromagnetic fields, is referred to as a Surface Plasmon Polariton (SPP) when it propagates along a planar interface, or a Localized Surface Plasmon (LSP) when it is restricted to a small, closed surface such as a subwavelength nanoparticle. These two regimes—propagating SPPs and localized LSPs—constitute the electromagnetic toolkit that metasurfaces exploit to confine light below the diffraction limit, a prerequisite for any surface-enhanced spectroscopy.

Building on these seminal insights, modern metasurfaces unify principles from historically distinct fields: frequency-selective surfaces (FSS), impedance sheets, and planar antenna arrays [8,11]. Unlike photonic crystals, these structures do not require strict, repeating arrangements [1]. Moreover, metastructures typically interact with light arriving from above or at an angle, whereas photonic crystals primarily rely on in-plane, bound propagating modes. In the optical regime, plasmonic metasurfaces exploit SPs for subwavelength light confinement, while dielectric metasurfaces leverage low-loss multipolar resonances, including electric and magnetic modes, to achieve efficient control over the light field [12]. Multipolar resonance refers to the excitation of higher-order modes (e.g., magnetic dipoles, electric quadrupoles) beyond the fundamental electric dipole response, enabling advanced manipulation of phase, directionality, and scattering. Compared to plasmonic metastructures, dielectric metasurfaces benefit from intrinsically lower absorption losses, especially at visible and near-infrared frequencies, making them more suitable for applications requiring high efficiency. Furthermore, they can exhibit “optical magnetism”, a phenomenon where nonmagnetic dielectric nanostructures mimic magnetic responses to light through the excitation of magnetic Mie-type resonances. This capability is particularly important because it allows for control of both the electric and magnetic components of light using compact, low-loss materials. Common materials for dielectric metasurfaces include silicon, germanium, and titanium dioxide, which offer high refractive indices and minimal optical losses across broad spectral windows, supporting both high-field confinement and precise phase modulation. Both approaches bridge the gap between Maxwell’s constitutive equations and practical photonic technologies, offering a cohesive framework for applications ranging from high-efficiency metalenses [4] to conformal radiators [13].

In addition to wavefront manipulation and imaging, metasurfaces have recently shown significant potential for enhancing spectroscopic techniques. Spectroscopy is a vital analytical technique used to study the composition of materials, for example, by analyzing how they scatter light, absorb infrared radiation, or emit fluorescence. This provides information about their molecular structure and properties [14]. By providing strong electromagnetic field localization at the nanoscale, resonant structures can boost the signal-to-noise ratio in methods such as Surface-Enhanced Raman Scattering (SERS), Surface-Enhanced Infrared Absorption (SEIRA), and Surface-enhanced fluorescence (SEF). However, traditional SERS, SEIRA, and SEF techniques can be limited in sensitivity, resolution, and reproducibility, thus compromising their ability to detect and analyze trace amounts of substances or complex molecular structures [15]. In recent years, metasurfaces have emerged as a promising alternative to overcome these problems. This has opened new avenues in chemical and biochemical sensing, environmental monitoring, and biomedical diagnostics, further underscoring the versatile capabilities of metasurface-based devices.

Three reviews have been published in the last five years to the best of our knowledge. The first, by Barbillon et al. (2022), focuses on the latest advances in the performance of plasmonic or dielectric metasurfaces for SERS and SEIRA sensors, as well as photocatalysis, highlighting enhancement factors ranging from 2 to 81 for photocatalysis, from 10 to $6\times 10^{3}$ for SEIRA sensors, and from $10^{4}$ to $3\times 10^{8}$ for SERS sensors [14]. Herpin et al. (2023) focused their review on recent developments in the field of metasurface-enhanced IR spectroscopy, highlighting that the integration with materials such as 2D van der Waals layers, microfluidics, and CMOS-compatible components is enabling compact, flexible, and high-performance sensor technologies for real-world applications [16]. They concluded that AI-driven analysis, spectrometerless detection, and on-chip integration promise transformative impact in fields ranging from biomedical diagnostics to environmental monitoring. More recently, Li et al. (2025) provided a comprehensive discussion of various biomedical spectral detection technologies enhanced by metasurfaces, demonstrating the advantages of metasurfaces in improving critical detection parameters (e.g., sensitivity, detection limits, and rapid biomolecule detection) and providing a detailed description of biomedical detection procedures associated with metasurfaces [17]. With the historical context and key resonant mechanisms established, we conclude this section by delineating the precise scope of the present review. This review provides a comprehensive overview of resonant phenomena in subwavelength particles and metasurfaces—both plasmonic and dielectric—in the context of SERS, SEIRA, and SEF. This review aims to provide a comprehensive overview of the theoretical background of resonant phenomena in subwavelength particles and metasurfaces, focusing on both plasmonic and dielectric types. We discuss the principles of electromagnetic resonance and how it is used for SERS/SEIRA/SEF and explore the recent advancements in using plasmonic and dielectric metasurfaces for different applications. Furthermore, we highlight the challenges and future directions in this rapidly evolving field, emphasising the potential for metasurfaces to revolutionise enhanced spectroscopic techniques and broaden their applications.

## 2. Theoretical Background

The physics of subwavelength scattering constitutes the foundation of surface-enhanced spectroscopy techniques and a broad range of nanophotonic applications. The following sections delve into three major surface-enhanced techniques: SERS, SEIRA, and SEF. Each method exploits distinct electromagnetic resonance mechanisms, such as LSPs or dielectric Mie resonances, to amplify weak optical signals from molecules near nanostructured surfaces. Differences in excitation, emission, and enhancement pathways are examined in detail, alongside the role of molecular orientation, surface selection rules, and distance-dependent field effects.

### 2.1. Electromagnetic Resonant Phenomena

**Figure 1:**
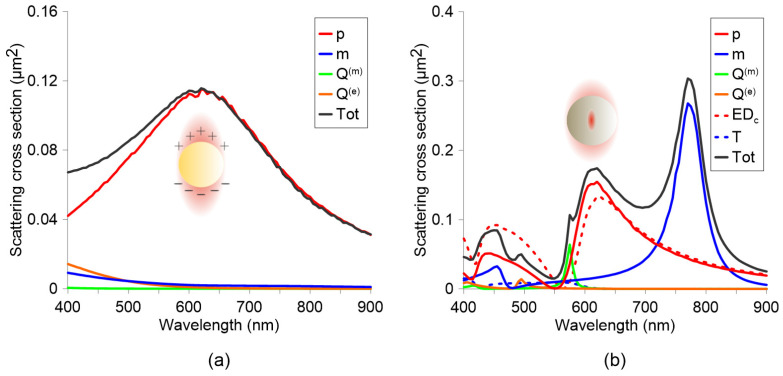
(**a**) Multipolar decomposition of the scattering cross-section of a 100 nm gold nanoparticle under plane-wave illumination and (**b**) multipolar decomposition of the scattering cross-section of a 100 nm silicon nanoparticle under the same conditions. The colored curves represent the individual contributions of different multipolar modes, including electric dipoles (p), magnetic dipoles (m), magnetic quadrupoles ($Q^{\left(m\right)}$), and electric quadrupoles ($Q^{\left(e\right)}$). In the case of the silicon nanoparticle, Cartesian electric dipole components (ED_c_s) and toroidal dipoles (Ts) are also included, shown as dashed lines. The black curve (Tot) indicates the total scattering cross-section. Results were obtained via COMSOL simulations.

At the beginning of the 20th century, Gustav Mie solved Maxwell’s equations to describe how light scatters off particles about the same size as the wavelength of light (for example, gold nanoparticles in water) [18]. Our understanding of scattering in metasurfaces stems from this foundation. The Mie solution accurately describes how a particle (with diameter ∼λ/n, *n* being the refractive index of the particle) scatters light, covering both the near-field and far-field regions [19]. The total scattering cross-section ($C_{\mathrm{sca}}$) is given by an infinite sum of spherical multipole terms, known as the Mie expansion:

(1) $$C_{\mathrm{sca}}=\frac{2\pi }{k^{2}}\sum_{m=1}^{\infty }\left(2m+1\right)\left(|a_{m}{|}^{2}+{\left|b_{m}\right|}^{2}\right),$$

where $a_{m}$ and $b_{m}$ are the electric and magnetic multipole coefficients of the *m*th order and *k* is the wavenumber. The Mie coefficients $a_{n}$ and $b_{n}$ can be obtained by solving Maxwell’s equations with boundary conditions at the surface of a sphere, resulting in expressions involving spherical Bessel and Hankel functions and their derivatives evaluated at the size parameter and refractive index contrast. They describe the scattering of the electric and magnetic components of the incident light at each multipole order. Another option is to use numerical methods, as shown in Figure 1. The multipolar decomposition of scattering in subwavelength particles is investigated by decomposing the fields inside the particles into spherical and Cartesian multipole moments [20]. This figure compares the numerically computed multipole expansions of the scattering cross-section for a 100 nm gold and silicon nanoparticle performed with COMSOL 6.0.

In the silicon nanoparticle case, both electric and magnetic modes emerge in the visible to near-infrared range, reflecting the significant contrast between the high refractive index of silicon and its surrounding medium. Near each resonance, the electromagnetic field becomes tightly confined within the particle, giving rise to strong Mie scattering and field enhancement often comparable to or exceeding that of plasmonic nanoparticles. In contrast, the gold nanoparticle exhibits a broad electric dipole peak that is dominated by metallic free-carrier absorption, leading to strong damping of the plasmonic mode. As a result, no pronounced magnetic dipole or higher-order magnetic multipoles appear for such a small gold sphere in the visible to near-infrared regime. The stronger dielectric response of silicon thus enables overlapping electric and magnetic Mie resonances, while gold’s lower refractive index contrast and intrinsic losses confine its dominant resonance to the electric dipole mode with significant dissipative broadening. The red and blue dashed curves in the silicon plot highlight how a conventional spherical electric dipole can be decomposed into Cartesian electric dipole and toroidal dipole components, which can interfere destructively and zero out the spherical electric dipole at a certain wavelength (∼550 nm in the case studied). The other colours represent different modes of the multipolar composition.

**Figure 2:**
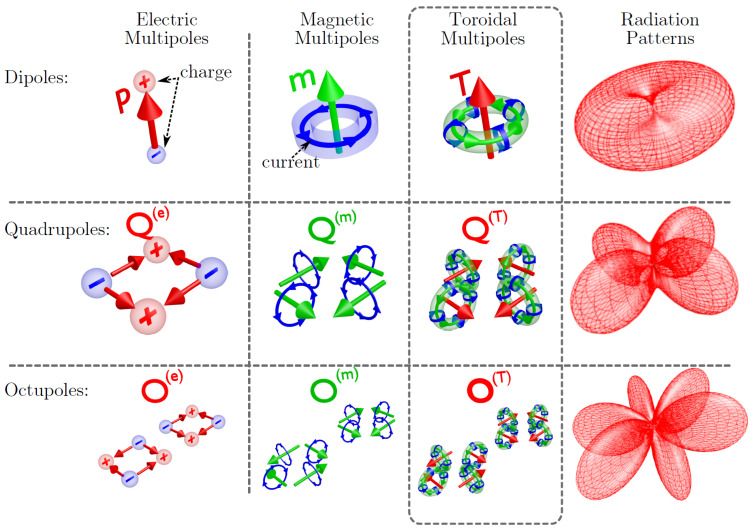
Three families of dynamic multipoles. The left columns show charge-current distributions for electric (p), magnetic (m), and toroidal (T) dipoles; electric ($Q^{\left(e\right)}$), magnetic ($Q^{\left(m\right)}$), and toroidal ($Q^{\left(T\right)}$) quadrupoles; and electric ($O^{\left(e\right)}$), magnetic ($O^{\left(m\right)}$), and toroidal ($O^{\left(T\right)}$) octupoles. The toroidal dipole (T) arises from poloidal currents flowing along a torus’s meridians. Anti-aligned toroidal dipoles and quadrupoles form $Q^{\left(T\right)}$ and $O^{\left(T\right)}$, respectively. The right column displays their radiation patterns. Reprinted with permission, © 2014 by the American Physical Society (APS).

Low-order (dipoles) and higher-order (quadrupole, octupoles, etc.) modes can be systematically categorized into three families (Figure 2): electric multipoles (*p*, $Q^{\left(e\right)}$, $O^{\left(e\right)}$), driven by oscillating charge densities; magnetic multipoles (*m*, $Q^{\left(m\right)}$, $O^{\left(m\right)}$), originating from circular displacement currents; and toroidal multipoles (*T*, $Q^{\left(T\right)}$, $O^{\left(T\right)}$), generated by poloidal currents such as the oscillating meridional flows on a torus that produce the toroidal dipole (*T*). Higher-order toroidal modes (e.g., $Q^{\left(T\right)}$, $O^{\left(T\right)}$) emerge from anti-aligned arrangements of lower-order counterparts; for example, a toroidal quadrupole comprises two oppositely oriented toroidal dipoles. The right column of Figure 2 illustrates the directional radiation patterns of these modes, which are critical for engineering metasurfaces with tailored angular responses such as beam steering or asymmetric scattering.

With the behavior of isolated nano-scatterers in hand, we now explore how ordering them into extended, sub-wavelength lattices fundamentally reshapes their optical response. Section 2.1.1 surveys plasmonic metasurfaces: arrays of gold, silver or aluminium nano-antennas whose coupled localized surface plasmon resonances (LSPRs) hybridize with diffractive channels to spawn hot spots, spoof–surface–plasmon polaritons at long wavelengths, and ultra-sharp surface–lattice resonances (SLRs) near Rayleigh anomalies. These collective modes funnel energy into nanometer gaps, produce extraordinary transmission or perfect absorption, and furnish the field enhancements that underpin single-molecule SERS and infrared chem-sensing. Section 2.1.2 then pivots to all-dielectric metasurfaces built from high-index silicon, germanium, or tellurium. Here, low-loss electric–magnetic Mie resonators support Huygens and Janus dipoles, toroidal currents and anapole states, whose interference can suppress back-scattering, steer wavefronts or trap light in quasi-bound states-in-the-continuum with record-high Q-factors. Multipole engineering, multimode coupling, and lattice symmetries thus provide two complementary yet equally powerful toolkits—plasmonic and dielectric—for sculpting dispersion, boosting near-fields, and enabling the advanced surface-enhanced spectroscopies treated in the remainder of this section.

#### 2.1.1. Plasmonic Metasurfaces

**Figure 3:**
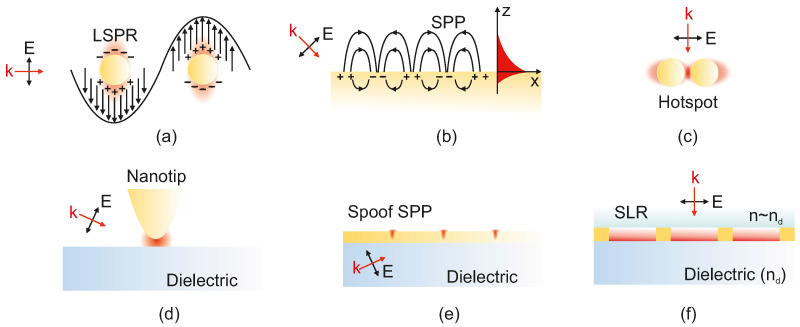
Plasmonic resonances. (**a**) Localized Surface Plasmon Resonances. (**b**) A nanometric metallic film supporting a Surface Plasmon Polariton. (**c**) Hot-spot between two nanoparticles. (**d**) A nanotip supporting localized Surface Plasmon Resonances and acting as a lightning rod. (**e**) A spoof SPP in a metal strip with periodic grooves. (**f**) A Surface Lattice Resonance in a periodic metasurface.

Localized surface plasmon resonances (LSPRs) are a phenomenon that occurs when conduction electrons on the surface of a metallic nanoparticle (usually gold or silver) oscillate collectively in response to incident light at a specific wavelength (see Figure 3a). These oscillations are “localized” because they are confined to the surface of individual nanoparticles, as opposed to extending across a bulk metal surface (SPP), Figure 3b. For instance, a spherical gold nanoparticle supports a localized plasmon resonance when Re[εm(ω)]≈−2εd and Im[εm(ω)] remains small [10,21]. Under these conditions, the enhancement of the near-field intensity can be enormous, which has driven seminal advancements in SERS and SEIRA. The design space for plasmonic materials spans a broad spectral range, from the visible and near-infrared (Au, Ag, Cu) [9,22] to the ultraviolet (Al, Ga, In, TiN) [9,23], and extends into the mid-infrared using III–V semiconductors or doped graphene [24]. Although reactive alkali metals (e.g., Li, Na, K) offer promising optical properties, their chemical reactivity poses stability challenges [23].

When two or more plasmonic nanoparticles are brought into close proximity—typically within a few nanometers—their individual LSPRs can couple, resulting in a concentrated electromagnetic field in the narrow gap between them. This interaction creates what is known as a hot-spot: a highly localized region where the near-field intensity is dramatically amplified beyond that of isolated nanoparticles (Figure 3c). These hot-spots are critically important in SERS, as molecules situated within them experience substantial enhancement of their Raman scattering signals; for example, dimers of closely spaced nanoparticles introduce sub-20 nm gaps that become hot-spots of pronounced field enhancement [25], enabling single-molecule SERS detection. For this reason, the ability to engineer and control such nanoscale gaps between plasmonic structures has thus become central to the design of high-sensitivity SERS substrates. Based on the same principle, tip-enhanced Raman scattering (TERS), Figure 3d, exploits sharp metallic tips in scanning probe geometries to achieve subwavelength resolution and strong local field enhancements [26,27].

In all cases, the geometry and arrangement of these metal nanostructures can be tailored to create metasurfaces that exhibit a variety of phenomena, including extraordinary optical transmission [28], perfect absorption [29], and intense electromagnetic field localization [30]. V-shaped antenna elements on silicon, for example, modify the scattering phase profile to impart orbital angular momentum onto transmitted beams [31]. Another example is the so-called Spoof SPPs, which can be realized at lower (e.g., microwave or terahertz) frequencies by corrugating conducting surfaces [24,32]. These engineered structures mimic the tight field confinement typical of optical plasmons, but in regimes where metals typically behave as perfect conductors and no natural plasmons exist. This concept is particularly attractive for mid-infrared applications such as SEIRA, where metallic antennas or patterned surfaces selectively enhance molecular vibrational signals. Another effect produced by the interplay of the localized modes and diffractive channels of the structure is Surface Lattice Resonances (SLRs), Figure 3f. Specifically, SLRs in plasmonic metasurfaces emerge from the coherent coupling between LSPRs of individual nanoparticles and the diffractive modes of the array. These resonances occur when the periodicity of the array satisfies specific phase-matching conditions with the incident light, typically near the Rayleigh anomaly. As a result, SLRs exhibit spectrally narrow linewidths and enhanced quality factors compared to isolated LSPRs, owing to the collective optical response of the array [9,23]. This interplay between localized and delocalized modes leads to highly tunable optical properties, enabling a broad range of capabilities for controlling and enhancing light at deeply subwavelength scales, and offering highly tunable platforms for biosensing, nonlinear optics, and quantum light–matter interfaces [33].

#### 2.1.2. Dielectric Metasurfaces

Dielectric metasurfaces, constructed from high-refractive-index materials such as silicon, germanium, or tellurium, leverage Mie resonances to generate and manipulate both electric and magnetic multipole responses [34,35]. Unlike plasmonic counterparts, they exhibit dramatically lower optical losses while still supporting electric and magnetic modes, making them highly attractive for subwavelength-scale applications in ultrathin optics and integrated photonics. These capabilities derive from three closely related design strategies that govern the control of resonances and interference pathways.

The first category involves multipole interference, where individual resonators can be viewed as meta-atoms or meta-molecules that support electric, magnetic, and more intricate current configurations such as toroidal moments. Huygens’ dipoles result from overlapping electric and magnetic dipoles that cancel backwards scattering, enabling near-unity transmission and unidirectional wavefront shaping following Kerker’s conditions [36]. Introducing a π/2 phase shift between these dipoles creates Janus dipoles, which break front–back symmetry to route light differently in each direction [37]. Then, the toroidal dipole is a type of Mie multipole that results from two circulating currents that run in opposite directions. Several applications with metasurfaces have been proposed [38,39]. Anapole states arise when electric and toroidal dipoles destructively interfere [40], suppressing far-field radiation so that energy remains in the near field [41]. These concepts of toroidal displacement currents and face-dependent scattering profiles illustrate how seemingly subtle arrangements of charges and currents produce strong directional effects, suppressed scattering, or enhanced near-field intensities.

A second category hinges on Mie resonance and non-trivial multimode coupling. Mie resonances emerge naturally in high-index nanoparticles once their dimensions approach the incident wavelength, yielding robust electric and magnetic dipole modes alongside higher-order excitations. This dual response enables precise tuning of scattering, absorption, and phase fronts. Complex interference of these modes can give rise to asymmetric Fano-type features and even quasi-bound states in the continuum (quasi-BIC), where destructive interference of radiative channels locks energy into long-lived modes with ultrahigh quality factors [42]. Few experimental works have been demonstrated at VIS and NIR due to the difficulty in obtaining perfect periodic structures and tolerances of EBL process [43]. Such Mie-driven phenomena allow strong control over far-field spectral characteristics and are crucial in sensing, lasing, and nonlinear light generation [44].

A final branch of design focuses on multimode coupling in extended metastructures, where collective effects in periodic or clustered arrays further enrich the range of achievable resonances. Meta-lattices or meta-assemblies can support SLRs featuring sharpened spectral lines and increased field enhancement. Arranging achiral or chiral building blocks yields novel light–matter interactions, including circular dichroism and polarization-sensitive response, while hybrid metastructures fusing dielectric resonators with plasmonic or excitonic components benefit from strong field confinement and tunable spectral overlap. These coupled modes expand opportunities for applications like subwavelength imaging, compact biosensors, and high-efficiency nonlinear devices by leveraging extreme near-field intensities and selective scattering pathways.

Taken together, these design strategies underscore the versatility and power of dielectric metasurfaces in achieving complex optical functionality through carefully engineered multipole interference, Mie resonance, and multimode coupling. The interplay of electric, magnetic, and toroidal modes, exemplified by anapole states or bound states in the continuum, provides strong field localization and low radiative losses for advanced photonic technologies. By harnessing these interference effects and coupling mechanisms, researchers continue to push the boundaries in wavefront shaping, ultrafast nonlinear optics, and enhanced spectroscopy, including Raman and infrared absorption-based sensing techniques. The result is a growing suite of metasurface architectures reviewed in Section 4.

### 2.2. Electromagnetic and Chemical Enhancement in SERS, SEIRA, and SEF

Surface-enhanced spectroscopy exploits the interaction of molecules with subwavelength structures (typically noble metals and dielectric materials) to greatly amplify weak optical signals. In this section, we provide the theoretical foundations of three such techniques: SERS, SEIRA, and SEF. We outline the electromagnetic mechanisms underlying these enhancements, as well as the role of molecular adsorption (chemical effects), accompanied by relevant equations and illustrative figures.

#### 2.2.1. SERS

SERS is based on the inelastic scattering of light, where incident photons interact with the vibrational or phonon modes of a molecule or material. This process involves two key steps: excitation of vibrational modes at a frequency $\omega_{\mathrm{ex}}$ followed by emission at a Stokes-shifted frequency $\omega_{\mathrm{em}}=\omega_{\mathrm{ex}}-\Omega$, where Ω represents the vibrational frequency. The Raman scattering intensity is proportional to the fourth power of the emission frequency and depends on the local electric field enhancement near the surface. SERS primarily benefits from mechanisms such as Mie resonances, the Purcell effect, and stored electric energy in nanostructures. These factors amplify the electromagnetic fields around the nanostructure, enhancing the Raman signal by orders of magnitude. Despite the advantages of SERS in molecular specificity and single-molecule sensitivity [45], practical implementation faces significant challenges, including fluorescence interference in biological media, nanofabrication complexities for reproducible hot-spot generation [46], and limited spectral tunability in conventional plasmonic systems. Metasurfaces overcome these constraints through precision engineering of optical resonances by controlling geometric parameters (size, lattice symmetry, unit cell design), optimizing material composition, and tailoring dielectric environments. This enables the rational design of substrates suited to specific application domains. In particular, the demands of biomedical Raman spectroscopy—such as biocompatibility, spectral selectivity, and fabrication consistency—are effectively addressed by metasurface architectures. These properties enable the development of SERS substrates that combine precise optical response with high structural reliability [47,48,49], establishing metasurfaces as superior platforms for next-generation biomedical sensing.

Electromagnetic Enhancement: From a theoretical perspective, the total enhancement observed in SERS arises from multiple contributing mechanisms, primarily categorized into electromagnetic enhancement and chemical enhancement. The dominant mechanism is electromagnetic (EM) enhancement, which results from the excitation of LSPRs in nanostructured metallic substrates, such as gold and silver. This effect significantly amplifies the local electromagnetic field, thereby increasing the intensity of the Raman signal. A convenient starting point for quantifying SERS is Placzek’s polarizability theory. In the presence of an enhanced electromagnetic field near a plasmonic nanostructure, the Raman intensity $I_{\mathrm{SERS}}^{k}$ of a particular vibrational mode *k* can be written as [50]

(2) $$I_{\mathrm{SERS}}^{k}=\frac{2^{7}}{3^{2}}\frac{\pi^{5}}{c^{4}}\;I_{0}{\left(\nu_{0}-\nu_{k,mn}\right)}^{4}\sum_{\rho \sigma }{\left|{\left(\alpha_{\rho \sigma }\right)}_{mn}\right|}^{2}N\;A\;\Omega \;Q\;T_{m}\;T_{0}\;\cdot \;G_{\mathrm{SERS}},$$

where $I_{0}$ is the incident intensity, $\nu_{0}$ and $\nu_{k,mn}$ (in cm^−1^) are the incident and $k^{th}$ vibrational-mode frequencies between the vibrational levels *m* and *n*, respectively, ${\left(\alpha_{\rho \sigma }\right)}_{mn}$ is the polarizability derivative, *N* is the surface number density of adsorbates, *A* is the laser-illuminated area, Ω is the solid angle of the collection optics, and $QT_{m}T_{0}$ is the product of detector efficiency and transmission factors.

**Figure 4:**
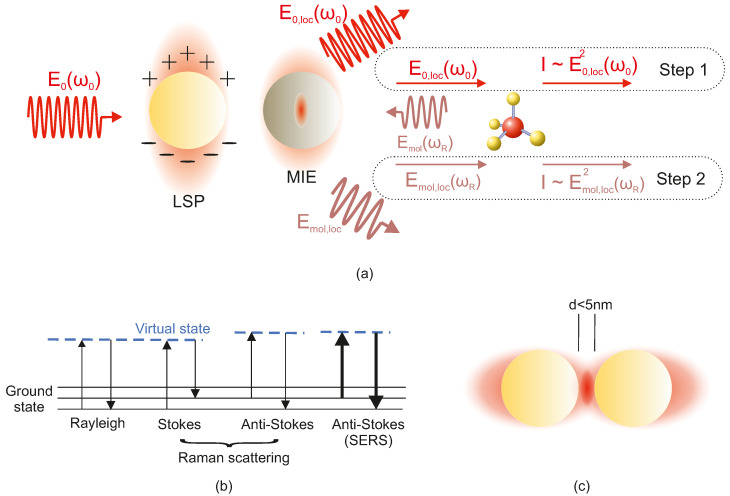
(**a**) Schematic of the two-step electromagnetic enhancement mechanism in SERS, illustrating both the LSP response and the Mie scattering contribution that boost the local electromagnetic field. (**b**) Jablonski diagram comparing ordinary Raman scattering (Stokes, anti-Stokes) with the additional enhancement achieved in SERS. (**c**) Illustration of two closely spaced nanoparticles (d < 5 nm) forming a hot-spot, where the local field is maximized for optimal SERS enhancement.

The term $G_{\mathrm{SERS}}$ is the overall SERS enhancement factor that can be calculated by considering the two-step approach (Figure 4) [51,52]:

1. Local field enhancement at the incident laser frequency $\omega_{0}$,
2. Radiation enhancement at the scattered (Raman) frequency $\omega_{R}$.

A simplified description starts by considering a molecule at position $r_{m}$ near a plasmonic nanostructure (“antenna”). We let $E_{0}\left(\omega_{0}\right)$ be the incident field (in free space) and $E_{\mathrm{loc}}\left(\omega_{0},r_{m}\right)$ be the local field near the nanostructure. We define the local-field enhancement factor as

(3) $$g_{1}\left(\omega_{0},r_{m}\right)=\frac{E_{\mathrm{loc}}\left(\omega_{0},r_{m}\right)}{E_{0}\left(\omega_{0}\right)}.$$

The induced dipole in the molecule at the Raman-shifted frequency $\omega_{R}$ is

(4) $$p_{m}\left(\omega_{R},r_{m}\right)=\alpha_{m}^{I}\left(\omega_{R},\omega_{0}\right)\;E_{\mathrm{loc}}\left(\omega_{0},r_{m}\right),$$

where $\alpha_{m}^{I}\left(\omega_{R},\omega_{0}\right)$ is the Raman polarizability tensor for the mode of interest. The total dipole contribution is the sum of the induced dipole and the antenna’s induced dipole

(5) $$p\left(\omega_{R}\right)=p_{m}\left(\omega_{R},r_{m}\right)+p_{A}\left(\omega_{R},r_{A}\right),$$

where the antenna’s dipole under induced dipole approximation is

(6) $$p_{A}\left(\omega_{R},r_{A}\right)=\omega_{R}^{2}\;\mu_{0}\;\alpha_{A}\left(\omega_{R},R\right)\cdot G_{A-m}\left(R,r_{m}\right)\cdot p_{m}\left(\omega_{R},r_{m}\right).$$

Then, the total dipole contribution is

(7) $$p\left(\omega_{R}\right)=\left(1+\omega_{R}^{2}\;\mu_{0}\;\alpha_{A}\left(\omega_{R},R\right)\cdot G_{A-m}\left(R,r_{m}\right)\right)\cdot p_{m}\left(\omega_{R},r_{m}\right),$$

(8) $$p\left(\omega_{R}\right)=g_{2}\left(\omega_{R},r_{A}\right)\cdot p_{m}\left(\omega_{R},r_{m}\right).$$

Then, the term $g_{2}\left(\omega_{R},r_{A}\right)$ corresponds to an emission enhancement factor, which can be interpreted as an additional “re-radiation” effect induced by the molecular dipole exciting the nanostructure

(9) $$g_{2}\left(\omega_{R},r_{A}\right)=1+\omega_{R}^{2}\;\mu_{0}\;\alpha_{A}\left(\omega_{R},R\right)\cdot G_{A-m}\left(R,r_{m}\right)=\frac{E_{\mathrm{mol}}\left(\omega_{R}\right)}{E_{\mathrm{mol},0}\left(\omega_{R}\right)},$$

where $\alpha_{A}\left(\omega_{R},R\right)$ is the antenna (nanostructure) polarizability and $G_{A-m}\left(R,r_{m}\right)$ is the dyadic Green’s function describing coupling between the molecule and the nanostructure.

Substituting Equations (3) and (4) into Equation (8) gives

(10) $$p\left(\omega_{R}\right)=g_{1}\left(\omega_{0},r_{m}\right)\cdot g_{2}\left(\omega_{R},r_{A}\right)\;\alpha_{m}^{I}\left(\omega_{R},\omega_{0}\right)\cdot E_{0}\left(\omega_{0}\right).$$

Noting that the scattered SERS intensity $I_{\mathrm{SERS}}\left(\omega_{R}\right)$ is proportional to $|p\left(\omega_{R}\right){|}^{2}$,

(11) $$I_{\mathrm{SERS}}\left(\omega_{R}\right)\propto {\left|g_{1}\left(\omega_{0},r_{m}\right)\right|}^{2}\;{\left|g_{2}\left(\omega_{R},r_{A}\right)\;\alpha_{m}^{I}\left(\omega_{R},\omega_{0}\right)\cdot E_{0}\left(\omega_{0}\right)\right|}^{2}.$$

In contrast, the normal Raman intensity $I_{\mathrm{NR}}\left(\omega_{R}\right)$ is

(12) $$I_{\mathrm{NR}}\left(\omega_{R}\right)\propto {\left|\alpha_{m}^{I}\left(\omega_{R},\omega_{0}\right)\cdot E_{0}\left(\omega_{0}\right)\right|}^{2}.$$

Hence, the electromagnetic SERS enhancement factor is [52]

(13) $$G_{\mathrm{EM}}=\frac{I_{\mathrm{SERS}}\left(\omega_{R}\right)}{I_{\mathrm{NR}}\left(\omega_{R}\right)}=|g_{1}\left(\omega_{0},r_{m}\right){|}^{2}\cdot {\left|g_{2}\left(\omega_{R},r_{A}\right)\right|}^{2}={\left|\frac{E_{\mathrm{loc}}\left(\omega_{0}\right)}{E_{0}\left(\omega_{0}\right)}\right|}^{2}\cdot {\left|\frac{E_{\mathrm{mol}}\left(\omega_{R}\right)}{E_{\mathrm{mol},0}\left(\omega_{R}\right)}\right|}^{2},$$

A common approximation near a strong plasmon resonance is $|g_{1}\left|∼\right|g_{2}|$, leading to the well-known “fourth-power law” ($|g_{1}{|}^{2}|g_{2}{|}^{2}\approx {\left|g_{1}\right|}^{4}$). This means that the total electromagnetic SERS enhancement often scales roughly as the local field enhancement to the fourth power. Classical treatments using Mie theory or finite element simulations of a molecule near a metallic sphere or in a nanoparticle junction predict these *E*-field amplification effects and reproduce typical SERS enhancement factors [50,53]. In practical terms, SERS intensity $I_{\mathrm{SERS}}$ for a molecule on a SERS-active substrate is related to the normal Raman intensity $I_{\mathrm{Raman}}$ (without enhancement) by an overall SERS enhancement factor EF:

(14) $$\mathrm{EF}\;=\;\frac{I_{\mathrm{SERS}}/N_{\mathrm{ads}}}{I_{\mathrm{Raman}}/N_{\mathrm{bulk}}}\;,$$

where $N_{\mathrm{ads}}$ is defined as the estimated number of molecules located within the effective hot-spot regions that contribute most strongly to the SERS signal, and $N_{\mathrm{bulk}}$ is the number of molecules probed in a conventional Raman setup [50,51]. This formulation is often used to quantify substrate performance. SERS EFs in the $10^{6}$–$10^{8}$ range are common for many roughened electrodes or colloidal nanoparticles, while specially engineered hot-spot substrates (e.g., aggregated colloids, nanoparticle dimers, or lithographic nanogaps) can achieve EFs $>10^{8}$ enabling single-molecule SERS detection [54].

Chemical Enhancement: Chemical enhancement in SERS originates from the intricate interplay between the adsorbed molecule and the substrate. This interaction induces changes in the electronic structure of the molecule, effectively modifying its polarizability and increasing the Raman cross-section [55]. Several mechanisms contribute to this phenomenon, including charge transfer between the molecule and the substrate, the formation of new electronic states at the molecule–metal interface, and modifications to the molecule’s polarizability [56]. When a molecule chemisorbs, new electronic states (hybrid orbitals between the metal and molecule) can form, resonantly enhancing Raman scattering by increasing the molecular polarizability α at the incident or scattered frequency. This mechanism is highly molecule-specific and typically contributes enhancements of 10–100-fold [52], much smaller than the EM effect but important for certain molecules that adsorb strongly. The chemical mechanism can be described quantum mechanically as a resonance Raman effect via metal-to-molecule charge-transfer states [57]. In practice, the highest SERS signals often occur when both mechanisms cooperate (e.g., an adsorbed dye molecule that has charge-transfer resonance on a plasmonic hot-spot). The overall SERS signal can thus be viewed as

(15) $$I_{\mathrm{SERS}}\;\approx \;I_{0}\cdot \sigma_{R}\cdot G_{\mathrm{EM}}\cdot G_{\mathrm{chem}}\;,$$

where $I_{0}$ is the incident intensity, $\sigma_{R}$ the normal Raman cross-section of the molecule, $G_{\mathrm{EM}}$ the electromagnetic enhancement factor, and $G_{\mathrm{chem}}$ the chemical enhancement factor (often $G_{\mathrm{chem}}∼10^{1}$–$10^{2}$ when active) [51,52].

Because the EM enhancement decays exponentially with distance from the resonant surface, SERS is extremely surface-sensitive. Only molecules in close proximity to the resonant structure (within a few nanometers) experience the intense field. This leads to the surface selection rules in SERS: for molecules on a flat metal surface, vibrational modes with polarizability changes perpendicular to the surface are enhanced the most. However, on nanoscale roughness, the local field can have components normal to the surface, relaxing the strict selection rule and allowing many vibrational modes to appear in SERS spectra (similar to normal Raman selection rules, but often with some orientation preferences).

For instance, studies have demonstrated that gold colloidal systems exhibit favorable performance in SERS analysis of amphetamine–metal interactions [58]. On the other hand, while charge transfer between the dielectric material and the analyte molecules contributes to the enhancement, it is not the sole mechanism [59]. Chemical enhancement in dielectric metasurfaces can also arise from changes in the electronic structure of the molecules due to their interaction with the dielectric material. The band gap of the dielectric material plays a crucial role in this process, influencing the charge transfer process and the overall SERS enhancement [59].

#### 2.2.2. SEIRA

SEIRA addresses the sensitivity limitations of conventional infrared spectroscopy, a label-free technique widely used to analyze molecular composition through vibrational fingerprints in biochemical systems. While infrared spectroscopy provides critical insights into chemical bonding and functional groups—particularly in the fingerprint region (600–1450 cm^−1^) and amide I/II regions (1500–1700 cm^−1^)—its effectiveness is constrained by weak vibrational absorption signals. This weakness stems from molecular vibration dipole moments being orders of magnitude smaller than infrared wavelengths, necessitating large biomolecule quantities for detectable signals [60]. SEIRA overcomes this limitation by coupling molecular vibrations with resonances in metallic or dielectric nanostructures, amplifying the optical near-field through plasmonic or Mie enhancement mechanisms. For example, the enhanced field strength at key infrared wavelengths (2550–3500 cm^−1^ for stretching vibrations like N–H and C–H, lower wavenumbers for bending/fingerprint vibrations) enables trace biomolecule detection [61,62]. Modern SEIRA platforms leverage metasurfaces to combine tailored near-field localization with advanced measurement configurations such as transmission, transflection, and attenuated total reflection (ATR) modes. These systems often employ focal plane array detectors or raster-scanning techniques for spatially resolved measurements [16]. A notable advancement involves metamaterial perfect absorbers (MPAs) in metal–insulator–metal (MIM) configurations, which demonstrate significantly enhanced absorption spectra compared to planar films, as shown by the improved detection of bovine serum albumin (BSA) on aluminium MPAs [63]. Critical to SEIRA’s performance is the precise spectral matching between resonances and molecular vibrational modes, exemplified by antenna–protein coupling models that replicate resonance offsets with the amide-II band at 1537 cm^−1^ [64]. Despite its advantages for studying chemical bonding and protein-binding interactions (e.g., surface coating efficiency and antibody enrichment [65]), SEIRA faces challenges including stringent substrate optimization requirements and lower trace sensitivity compared to SERS. Recent advances in nonequilibrium Green’s function formalism for modelling light-driven antenna systems and metasurface-enabled resonance tuning suggest promising solutions [63]. These developments position SEIRA-enhanced metasurfaces as transformative tools for biomedical spectroscopy, particularly for analyzing complex vibrational signatures in the biologically critical fingerprint region [66,67,68].

Electromagnetic Enhancement: The primary mechanism in SEIRA is again electromagnetic enhancement. Resonant nanostructures (or continuous metal films with appropriate nanoscopic roughness or islands) support LSP or Mie even at IR frequencies (often called “antenna resonances” in the IR). When the frequency of incident IR light $\omega_{\mathrm{IR}}$ matches or is near the plasmon resonance of the metal structure, the local electric field at the surface is intensified, boosting the absorption by nearby molecular vibrations [50]. In classical terms, the molecule’s dipole absorption strength is increased by the factor $|E_{\mathrm{loc}}\left(\omega_{\mathrm{IR}}\right){|}^{2}$ in the presence of the metallic nanostructure. The absorbance A(ω) (proportional to the imaginary part of the molecular polarization) can be written as

(16) $$A\left(\omega \right)\;\propto \;N\;|E_{\mathrm{loc}}{\left(\omega \right)|}^{2}\;\sigma_{\mathrm{abs}}\left(\omega \right)\;,$$

where *N* is the number of molecules and $\sigma_{\mathrm{abs}}$ is the IR absorption cross-section of the molecule. Note that this expression represents the dependence of the absorbance on the local field intensity. By comparison, without the resonant structure, $A_{0}\left(\omega \right)\propto N\;{\left|E_{0}\right|}^{2}\sigma_{\mathrm{abs}}$. Thus, unlike SERS, which benefits from both incident-field enhancement and re-radiation at a different frequency, SEIRA primarily depends on a single local-field enhancement at the absorption frequency $\omega_{k}$. Thus,

(17) $$G_{\mathrm{SEIRA}}={\left|\frac{E_{\mathrm{loc}}\left(\omega_{k}\right)}{E_{0}\left(\omega_{k}\right)}\right|}^{2}.$$

Owing to this one-step mechanism, SEIRA enhancement factors are often smaller than their SERS counterparts. Also, IR plasmons typically do not confine fields as tightly as visible-light plasmons (due to the longer wavelengths); the achievable ${\left|\;E_{\mathrm{loc}}\right|}^{2}$ enhancements are more modest, yielding $G_{\mathrm{SEIRA}}$ on the order of 10 to $10^{3}$. Nevertheless, this is enough to detect vibrational bands of monolayers that would otherwise be too weak. It is important to note that this expression refers to the local enhancement experienced by molecules located in hot spots. When estimating measurable absorbance enhancement over a macroscopic beam area, one must also consider the effective area covered by these hot spots relative to the total illuminated area. This is essential to obtain realistic estimation of signal enhancement, as is also the case for Equation (15). SEIRA-active substrates commonly include nanoantennas, metal island films, or porous nano-structured metals resonant in the mid-IR [69]. Modern designs use lithographically patterned metasurfaces tuned to specific IR vibrational frequencies to maximize $E_{\mathrm{loc}}$ at those frequencies, thereby maximizing absorption of target molecular vibrational modes [50].

Apart from enhancing intensity, an important aspect of SEIRA is the surface selection rule. Similar to molecules on a metal surface in IR reflection–absorption spectroscopy, only vibrational modes with a dipole moment component normal to the metal surface are significantly enhanced (or even observable) [69]. Vibrations oriented parallel to the metal surface produce oscillating dipoles that do not radiate efficiently into the far field. Therefore, SEIRA tends to highlight vibrations with transition dipoles perpendicular to the substrate. On highly nanostructured surfaces (e.g., particle clusters), this rule can be somewhat relaxed due to the complex field distribution, but it still influences the relative SEIRA band intensities [52]. This selection rule is the IR analogue of SERS surface selection rules for polarizability—both stem from the electromagnetic boundary conditions at a metal surface.

Chemical Enhancement: Chemical effects in SEIRA are generally minor but can occur if adsorption to the metal changes a molecule’s intrinsic IR absorption (for instance, via electronic effects or specific binding that alters force constants), which can lead to frequency shifts or intensity changes in IR bands. However, such chemical effects do not usually provide large uniform enhancement of all bands; rather, they might enhance certain modes via a coordination-induced increase in dipole moment. In most analyses, the observed SEIRA enhancement is attributed predominantly to the EM effect (field concentration), with any chemical contributions considered secondary [50,52].

#### 2.2.3. SEF

SEF is a powerful spectroscopic technique that significantly amplifies the fluorescence emission of fluorophores located in proximity to nanostructured surfaces [70]. While traditionally achieved using metallic surfaces and often referred to as metal-enhanced fluorescence (MEF), recent advances have demonstrated that SEF can also be realized with dielectric nanostructures, expanding the scope and versatility of this technique [71]. SEF offers a means to overcome the limitations of conventional fluorescence spectroscopy, such as photobleaching and relatively low signal intensity, particularly in applications requiring high sensitivity, like single-molecule detection and bioimaging [72,73]. This reduced photobleaching susceptibility arises from enhanced excitation efficiency and shortened excited-state lifetimes due to radiative rate enhancement (Purcell effect), which together lower the overall photon exposure of the fluorophore. In the case of metallic nanostructures, SEF arises primarily from the interaction between the excited state of a fluorophore and the LSP. This interaction can lead to enhanced excitation rates, modified emission lifetimes, and increased quantum yields, ultimately resulting in a dramatic increase in the observed fluorescence intensity [74,75]. In dielectric nanostructures, such as silicon or titanium dioxide, the enhancement is attributed to the excitation of optical resonances, such as Mie resonances [76,77]. These resonances can also create localized regions of high electric field intensity, although typically not as strong as plasmonic fields, that enhance the fluorophore excitation and emission rates. The underlying mechanisms of SEF are multifaceted and depend on factors such as the size, shape, and composition of the nanostructures (both metallic and dielectric), the distance between the fluorophore and the nanostructure, and the spectral overlap between the fluorophore emission and the optical resonances of the nanostructure [78]. When a fluorophore is placed near a resonant nanostructure, the oscillating electromagnetic field can enhance its excitation rate. Additionally, the nanostructure can act as a nanoantenna, efficiently coupling the emitted fluorescence into the far field, thereby increasing the collection efficiency [79]. The interplay between these effects can lead to fluorescence enhancements of several orders of magnitude, although the specific enhancement factors differ between metallic and dielectric platforms [80]. SEF substrates, both metallic and dielectric, are typically fabricated using techniques such as electron-beam lithography, nanosphere lithography, or chemical synthesis, allowing for precise control over the morphology and optical properties of the nanostructures [81,82]. Various metallic materials, including gold, silver, and aluminium, have been employed in SEF, each offering unique advantages in terms of plasmon resonance wavelength, biocompatibility, and chemical stability [83]. Similarly, dielectric materials such as silicon, titanium dioxide, and silicon nitride offer a range of refractive indices and fabrication possibilities, enabling tailored optical resonances and functionalities [84]. The choice of material and nanostructure design is crucial for optimizing the SEF effect for a specific fluorophore and application. SEF has found widespread applications in various fields, including biosensing, bioimaging, and medical diagnostics [73,85]. For instance, SEF-based biosensors have been developed for the highly sensitive detection of DNA, proteins, and other biomolecules [86,87]. In bioimaging, SEF enables enhanced visualization of cellular structures and processes with improved contrast and resolution [88]. Furthermore, SEF has been explored for enhancing the performance of optoelectronic devices, such as light-emitting diodes and solar cells [89]. While metallic nanostructures have traditionally been the focus, dielectric metasurfaces are gaining increasing attention due to their lower losses, the potential for higher enhancement factors in specific spectral ranges, and CMOS compatibility [90]. Despite the significant advancements in SEF, certain challenges remain. For metallic SEF, these include the need for precise control over the fluorophore–nanostructure distance to avoid fluorescence quenching and the potential for metal-induced phototoxicity in biological applications [91,92]. For dielectric SEF, challenges include achieving sufficiently strong field enhancements compared to plasmonic counterparts and optimizing designs for specific fluorophores. Ongoing research is focused on addressing these challenges through advanced nanofabrication techniques, novel materials, and a deeper understanding of the fundamental mechanisms governing SEF, both in metallic and dielectric systems [93]. The continued development of SEF, encompassing both plasmonic and dielectric approaches, promises to further expand its applications in diverse fields, offering unprecedented sensitivity and control over light–matter interactions at the nanoscale.

**Figure 5:**
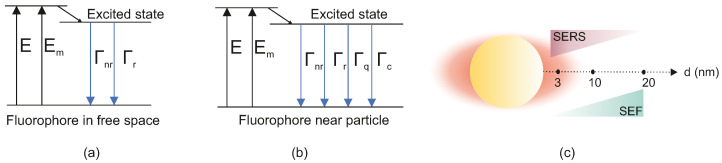
(**a**) Energy-level diagram of a free-space fluorophore, dominated by radiative ($\Gamma_{r}$) and non-radiative ($\Gamma_{nr}$) decay. (**b**) Fluorophore near a metal nanoparticle gains new decay channels—quenching ($\Gamma_{q}$) and enhanced radiative coupling ($\Gamma_{c}$)—altering its emission behavior. (**c**) Schematic illustration of the distance-dependent regimes for SERS (strongest under a few nanometers from the metal) and SEF (optimal at intermediate distances).

Electromagnetic Enhancement: There are two primary electromagnetic effects that a metal nanostructure has on a nearby fluorophore: (1) an enhanced local excitation field (the same $E_{\mathrm{loc}}$ effect that drives SERS) and (2) an alteration of the fluorophore’s radiative and non-radiative decay rates due to coupling with resonances (a Purcell effect). The excitation enhancement means that the fluorophore’s absorption of the excitation light is increased by ${\left|\;E_{\mathrm{loc}}\left(\omega_{\mathrm{exc}}\right)\right|}^{2}$, boosting the initial excited-state population. Meanwhile, if the emission frequency $\omega_{\mathrm{em}}$ is near a resonance, the fluorophore can couple to the nanoparticle and radiate its energy more efficiently (as plasmon-mediated radiation), effectively increasing its radiative decay rate $\Gamma_{r}$. However, energy transfer to the nanoparticle can also open a non-radiative decay channel (dissipating energy as heat in the metal), increasing the non-radiative rate $\Gamma_{\mathrm{nr}}$. In Figure 5a,b, the energy-level diagrams compare the fate of an excited fluorophore’s energy in free space (a) versus in the near-field of a nanostructure (b). In free space, the main channels for de-excitation are radiative emission ($\Gamma_{r}$) and intrinsic non-radiative decay ($\Gamma_{nr}$). When a fluorophore is brought close to a nanoparticle, additional decay pathways become possible—namely quenching or energy transfer to the nanoparticle ($\Gamma_{q}$) and enhanced radiative emission ($\Gamma_{c}$). These new channels shift the balance between radiative and non-radiative processes, enabling the fluorescence intensity to increase or decrease depending on the exact fluorophore–nanoparticle distance. Then, the fluorescent quantum yield Φ of a molecule is defined as $\Phi =\Gamma_{r}/\left(\Gamma_{r}+\Gamma_{\mathrm{nr}}\right)$ for its excited state. In the presence of a metal, this becomes

$$\Phi_{\mathrm{eff}}\;=\;\frac{\Gamma_{r}+\Gamma_{c}}{\Gamma_{r}+\Gamma_{\mathrm{nr}}+\Gamma_{c}+\Gamma_{q}},$$

An ideal SEF scenario is when $\Gamma_{c}$ is significant (to boost radiative emission) while $\Gamma_{q}$ remains small (to avoid quenching). The total fluorescence intensity $I_{\mathrm{fl}}$ can be written as

(18) $$I_{\mathrm{fl}}\;=\;I_{0}\;\sigma_{\mathrm{abs}}\;\Phi_{\mathrm{eff}}\;\propto \;{\left|\;E_{\mathrm{loc}}\left(\omega_{\mathrm{exc}}\right)\right|}^{2}\;\frac{\Gamma_{r}+\Gamma_{c}}{\Gamma_{r}+\Gamma_{\mathrm{nr}}+\Gamma_{c}+\Gamma_{q}}\;,$$

where $I_{0}$ is the incident intensity and $\sigma_{\mathrm{abs}}$ the absorption cross-section of the fluorophore. The term ${\left|\;E_{\mathrm{loc}}\right|}^{2}$ represents the excitation enhancement, and the fraction represents the modified quantum yield.

As shown schematically in Figure 5c, a molecule directly on a metal surface favors SERS (strong Raman, quenched fluorescence), whereas a molecule at a slight distance favors SEF (strong fluorescence, modest Raman). Unlike SERS, which benefits from ever-closer proximity of molecules to the metal, SEF typically has an optimal distance. If the fluorophore is too close (within ∼5 nm of the metal surface), $\Gamma_{\mathrm{nr}}^{\left(m\right)}$ (non-radiative energy transfer to the metal) becomes dominant, quenching fluorescence. If the fluorophore is too far (more than 20–30 nm), the local field enhancement is negligible and the metal has little effect. At an intermediate distance (often around 10–15 nm), one can obtain net enhancement of fluorescence: the excitation is boosted and the radiative decay is sped up, while quenching is mitigated [51]. This results in brighter emission than the molecule would have in free space. Metallic nanostructures can also direct the emission, enhancing detection efficiency by channeling the emitted light into specific angular distributions [73]. This competitive behavior is a hallmark of SEF theory. Quantitatively, enhancements in fluorescence intensity of up to 10-fold to 100-fold are commonly reported under optimal conditions [51], and in some experimental configurations, even higher factors (hundreds-fold) have been achieved. These are smaller than typical SERS gains because fluorescence already has a high base intensity (compared to Raman) and because of the quenching constraint. It is worth noting that the metal not only affects intensity but can also modify the fluorescence lifetime and emission spectrum. The decrease in fluorescence lifetime (due to increased radiative decay rate) is often observed as part of SEF and is consistent with the Purcell effect for emitters in optical cavities. Additionally, plasmonic surfaces can influence the emission directivity and polarization of the fluorescence. Additionally, plasmonic surfaces can influence the emission directivity and polarization of the fluorescence. While these effects are predicted in SEF theory, their strength and angular distribution are strongly influenced by engineering parameters such as metasurface geometry and dielectric environment. From the theoretical standpoint, the key point is that the presence of a plasmonic nanostructure alters the rates of excitation and de-excitation of the fluorophore.

## 3. Plasmonic Metasurfaces for Enhanced Spectroscopy

Plasmonic metasurfaces have been extensively studied for SERS, SEIRA, and SEF applications in recent years. These metasurfaces typically consist of periodic arrays of metallic nanostructures, such as nanoparticles, nanorods, or nanoantennas, which support strong localized electromagnetic fields. This field enhancement significantly amplifies spectroscopic signals from analytes positioned on or near the nanostructures, thereby enabling highly sensitive detection and analysis. Thus, plasmonic metasurfaces hold great promise for a broad range of advanced sensing applications [94]. In the field of biosensing, plasmonic metasurfaces have been widely employed to enhance the interaction between light and biomolecules through near-field confinement effects [95]. When combined with biofunctionalisation techniques, plasmonic metasurfaces present a promising approach for advancing biomarker detection technologies. They can facilitate both labelled and label-free biosensing methods. In labelled biosensing, plasmonic surface-enhanced detection techniques can significantly amplify the optical signals in fluorescence-based measurements. Conversely, label-free plasmonic biosensing has attracted increasing interest due to its potential for developing environmentally friendly, portable devices, highlighting its suitability for point-of-care testing applications [96].

In the following subsections, we explore recent advancements in plasmonic metasurfaces for each case: SERS, SEIRA, and SEF. We discuss how these innovations have contributed to enhancing the performance and applicability of these techniques, highlighting the latest developments and challenges in the field.

### 3.1. SERS

Several studies have demonstrated the effectiveness of plasmonic metasurfaces in enhancing SERS signals. As previously discussed, this phenomenon relies on the amplification of Raman signals due to the excitation of surface plasmons in nanoscale structures of noble metals, such as gold and silver. The optimization of plasmonic metasurface designs has led to the development of substrates with high enhancement efficiency and broad applicability, including trace-level molecular detection and biomedical diagnostics. However, one of the main challenges in integrating these metasurfaces into practical applications is achieving large-scale manufacturing. In this regard, Murthy et al. (2017) demonstrated scalable fabrication techniques, such as roll-to-roll extrusion coating, which enable the mass production of plasmonic metallic nanolayers with improved structural tolerance [97]. Also, increasing emphasis has been placed on the sustainability and scalability of fabrication processes. Karabel et al. (2019) developed eco-friendly techniques for synthesising plasmonic nanomaterials by grafting polyethylene glycol (PEG) layers onto solid substrates, simplifying gold nanoparticle immobilization while reducing the environmental impact of the process [98]. Complementarily, wet-chemical etching methods have enabled the fabrication of random plasmonic metasurfaces, providing cost-effective and highly reproducible solutions for SERS applications [99]. Recently, template-based electrodeposition techniques have demonstrated a cost-effective and reproducible alternative for the large-scale fabrication of plasmonic metasurfaces aimed at commercial applications in biomolecular detection [100].

In turn, several strategies have been implemented to enhance the efficiency of SERS substrates through innovative approaches in the generation of hot spots. For instance, Sakir et al. (2017) demonstrated the effectiveness of controlled silver nanocrystal growth in forming highly intense hot spots, achieving an enhancement factor of $1.3\cdot 10^{7}$ [101]. Thrift et al. (2017) employed electrohydrodynamic techniques to manipulate nanoparticles and create assemblies with sub-nanometric interparticle gaps, thereby optimizing the spectral response of SERS [102]. Additionally, hybrid structures combining gold and silver nanostructures have shown great potential for maximizing light capture and enhancing Raman signal uniformity, which is particularly beneficial for applications such as drug detection and chemical analysis, as demonstrated by Gao et al. (2018) [46].

**Figure 6:**
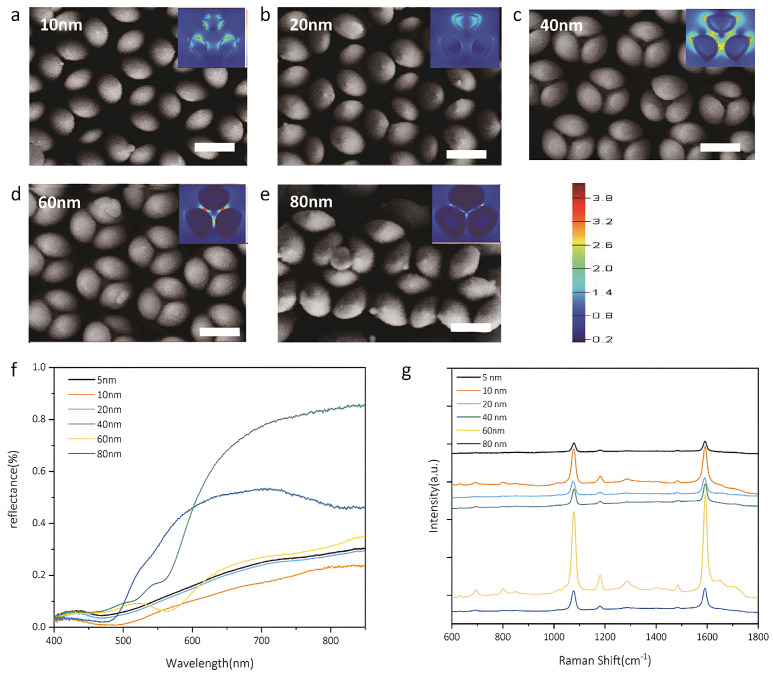
(**a**–**e**) SEM images of a nanoflower array with a gold layer thickness of 10, 20, 40, 60, 80 nm, respectively. All scales in the figure indicate 150 nm. The insets show FDTD-simulated maps of the normalized electric-field magnitude taken in the equatorial XY plane of each nanoflower under normal incidence of a 633 nm plane wave. (**f**) Reflectance spectra of nanoflower arrays of various gold layer thicknesses. (**g**) The SERS spectra of benzoic acid (1 × 10^−3^ M) on the nanoflower array. Reprinted with permission from [103]. Copyright 2019 John Wiley and Sons.

In addition to advancements in materials and geometries, three-dimensional architectures, such as the nanoflower-like metasurfaces proposed by Jiang et al. (2020), have generated multiple hot spots, significantly enhancing biomolecule detection sensitivity with an EF of approximately $10^{6}$ (Figure 6) [103].

Following a bioinspired approach, Narasimhan et al. (2020) developed flexible and disordered metal–insulator–metal (MIM) metasurfaces composed of Au–SiO_2_–Au layers on a PDMS substrate [104]. These structures were tested for the detection of uric acid in human tears, demonstrating their potential for portable biosensing and on-site diagnostics. Alternatively, Sarychev et al. (2020) initially investigated the anomalous optical response of metasurfaces composed of regular silicon resonators in the form of two-dimensional periodic bars coated with nanometer-thin silver films [105]. Continuing their investigations, they further advanced the field of SERS by developing holographic metasurfaces consisting of metal nanogratings fabricated on a dielectric substrate via optical interference lithography. These structures enable strong plasmonic localization, resulting in highly efficient and selective SERS sensors suitable for high-precision biomedical and chemical analysis, reaching a limit of detection (LoD) of 230 nM for 4-mercaptophenylboronic acid [30]. Focusing on alternative structural designs, Palermo et al. (2021) introduced pyramidal nanoslits in plasmonic metasurfaces, achieving ultrasensitive detection of the hepatitis A virus with detection limits in the picogram per millilitre range [106]. Regarding other strategies to increase the electric field, the incorporation of an underlying metallic layer in metal film over nanosphere (MFON) structures has significantly enhanced the SERS effect, increasing the electric field concentration in hot spots by up to fivefold [107]. Such innovations in plasmonic metasurfaces contribute to developing more efficient, scalable, and versatile SERS-based sensing technologies.

**Figure 7:**
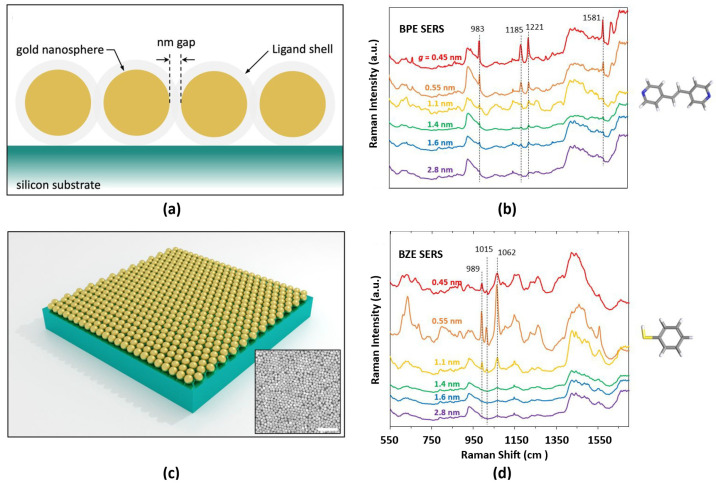
Depiction of Au nanosphere self-assembled metasurface on a Si substrate. (**a**) Cross-sectional view with Au nanospheres, nanoscale gaps, ligand shells, and Si substrate labelled. (**c**) Three-dimensional schematic of the resulting metasurface (not to scale and zoomed to show Au particles approaching all substrate edges, which may not necessarily occur during fabrication). Inset is a TEM image of a metasurface with 0.55 nm gaps (100 nm scale bar). And waterfall-plotted SERS spectra for a range of metasurface gap values from 0.45 to 2.8 nm. Enhanced Stokes shift peaks were observed for samples containing (**b**) trans-1,2-bis(4-pyridyl)-ethylene (BPE) and (**d**) benzenethiol (BZT). Reprinted with permission from [110]. Copyright 2022 American Chemical Society.

Developments in plasmonic metasurfaces for SERS have led to significant advancements in the control and amplification of electromagnetic fields. Structural engineering has led to the development of anisotropic metasurfaces with sub-wavelength grooves, being tunable SERS substrate and enabling precise directional control of the electromagnetic field, which is particularly valuable for two-dimensional materials such as graphene [108]. Another notable advancement is the integration of double Fano resonances in dimeric nanorings, which allows flexible tuning of resonance frequencies by adjusting the periodicity of the gold nanoring dimer array to enhance spectroscopic sensitivity [109]. In this context, Bauman et al. (2022) reported the implementation of gold nanosphere metasurfaces with tunable subnanometer gaps widths, achieving a field enhancement on the order of 10^4^ over measurements on bare Si (Figure 7) [110].

More recently, in the realm of tunable plasmonic surfaces, Marques et al. (2024) developed pyramidal metasurfaces that enable precise control over localized plasmon–resonant mode interactions, offering high sensitivity for biomolecule detection and holding promise for advanced biosensing applications [111]. They fabricated an all-metal pyramidal metasurface, which was tested for the detection of the Pb27r protein from *Paracoccidioides brasiliensis*, demonstrating a sensitivity approximately $10^{3}$ times higher than that of conventional LSPR-based sensors.

Beyond these structural advancements, the integration of artificial intelligence has expanded the analytical capabilities of SERS. Li et al. (2022) developed a system based on plasmonic nanocubes that, through deep learning, allows for the simultaneous analysis of multiple organophosphorus pesticides in water with high precision and speed reaching the single-molecule detection [112]. Additionally, Rippa et al. (2022) introduced a metasurface of three triangular hybrid Au–polymer octupolar nanocavities for detecting bacterial toxins such as Shiga toxins, achieving SERS enhancements of up to $9\cdot 10^{7}$, marking a significant advancement in biosensor technology [113].

Innovations in plasmonic metasurfaces have addressed various challenges in ultrasensitive detection through SERS. A key approach has been the development of reusable plasmonic sensors by incorporating DNA-based antiadhesive layers, enabling the regeneration of the active surface without compromising performance. This advancement, led by Trojanowicz et al. (2022), represents a promising solution for producing long-lasting SERS sensors with enhanced stability and low operational costs [114]. In this pursuit of more reliable sensors, Zeng et al. (2022) proposed optimizing a nanoparticle-on-metal metasurface based on a colloidal Langmuir–Schaefer deposition specifically designed for molecule-oriented detection, achieving precise alignment within nanogaps and significantly reducing the typical variability in Raman signals [49]. Moreover, Reyes-Coronado et al. (2022) investigated both experimentally and theoretically the enhanced light absorption in a supported, disordered plasmonic monolayer composed of gold nanospheres under a total internal reflection configuration [115]. These disordered metasurfaces, consisting of a random monolayer of Au nanospheres, were designed to optimize light absorption by leveraging leaky lossy guided modes within the monolayer, which result from a balanced interplay between near-field coupling and scattering of the nanoparticles.

Other studies focus on phenomena such as chirality to achieve strong, uniform, and reproducible Raman enhancement, even for achiral molecules. Jones et al. (2023) demonstrated that dense arrays of metallic nanohelices—fabricated via nano-glancing angle deposition—can generate strong near-field enhancements under circularly polarized light, significantly improving SERS signals from achiral analytes like crystal violet. Their nanohelical metasurfaces, composed of Au or Ag, exhibited clear circular intensity difference (CID) responses depending on the handedness of both the helices and the incident light, revealing a high degree of circular polarization sensitivity and excellent reproducibility, with standard deviations below 4% for Ag structures under optimized conditions [116]. Additionally, Xiao et al. (2023) reported a surface-enhanced Raman polarization rotation effect arising from the interaction between optically inactive molecules and the chiral plasmonic response of anisotropic gold metasurfaces. Their “nanoclock”-shaped structures enabled the generation of elliptically polarized Raman signals from linearly polarized excitation, effectively mimicking Raman optical activity (ROA) without requiring molecular chirality, and offering a robust strategy for probing Raman-inactive molecules with enhanced sensitivity [117].

**Figure 8:**
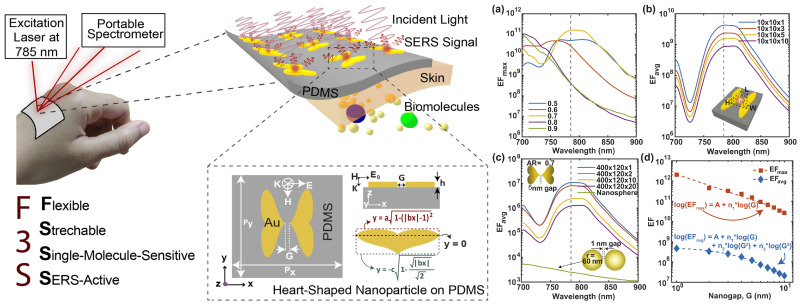
Schematic representation of the flexible plasmonic SERS sensor proposed by Haque et al., designed for real-time, label-free biochemical analysis of sweat using a portable Raman spectrometer. The main illustration shows the sensor laminated onto the wrist. The top inset highlights the heart-shaped gold nanodimer array embedded in a PDMS substrate, tailored for sweat sampling. The bottom inset presents the geometric model of the heart-shaped nanoparticle (NP), where *G* denotes the nanogap between dimers, $P_{x}$ and $P_{y}$ indicate the periodicity along the x- and y-axes, respectively, and *h* is the NP height along the z-axis. (**a**) Maximum enhancement factor (EF) as a function of wavelength for varying aspect ratios. (**b**,**c**) Average EF across different volumes for an aspect ratio of 0.7 at a wavelength of 785 nm. (**d**) Log-log plots of maximum and average EF versus nanogap *G* at 785 nm. Reproduced from [118], Royal Society of Chemistry. This article is licensed under a Creative Commons Attribution 3.0 Unported Licence.

Furthermore, the development of flexible and stretchable sensors has propelled SERS technology toward integration with portable devices for real-time biosensing. Haque et al. (2023) introduced a flexible, stretchable, and single-molecule-sensitive SERS-active (F3S) sensor based on a heart-shaped gold nanodimer array fabricated on a biocompatible PDMS substrate [118]. This plasmonic metasurface demonstrates an unprecedented combination of high SERS enhancement (up to 10¹¹), large hot-spot volume, and mechanical robustness under significant deformation, making it particularly well suited for wearable and in vivo applications. As illustrated in Figure 8, the sensor can be laminated directly onto the skin, enabling label-free biochemical analysis of sweat using a portable Raman spectrometer. Numerical simulations confirmed consistent SERS performance under bending up to 100° and stretching up to 50%, with only a minimal decrease in the enhancement factor. It is important to note that these results are based solely on simulations, and experimental validation of the sensor’s performance under such mechanical strain remains an open challenge. Notably, Figure 8 highlights the device’s maximum and average enhancement factors at various nanogap sizes and volume scales, showing that even with a relatively large 5 nm gap, the metasurface achieves SERS performance far exceeding that of conventional nanostructures (i.e., the widely used sphere, rod, disk, cube, void or mesh-based dimers/arrays). These results underscore the potential of the sensor in personalized medicine, offering real-time, non-invasive biomarker detection with single-molecule sensitivity in a mechanically flexible and scalable platform.

Advances in plasmonic metasurfaces have significantly expanded their capabilities for SERS applications, with a focus on hybrid designs, novel fabrication methods, and real-world implementations. For example, Kovalets et al. (2022) developed scalable fabrication techniques using stretch-induced microcracks on metalised membranes [119] and, more recently, Vickers indenter-based microscratching on PET films. This latter method enables precise control over hot-spot density, correlating SERS signal strength with microscratch geometry for reliable, reproducible enhancement [120].

On the application side, Trang et al. (2024) designed a portable SERS sensor within a centrifuge tube, using self-assembled Au-Ag heterodimers to form dense plasmonic hot-spots. The system achieved an enhancement factor of $4.69\times 10^{8}$ and enabled ultrasensitive detection of pesticide residues at concentrations as low as $10^{-8}\;M$, demonstrating strong reproducibility and suitability for on-site environmental monitoring [121]. In the biomedical domain, Zheng et al. (2024) developed a multiplexed SERS biosensing platform using gold–silica pyramidal metasurfaces that simultaneously enhance electric and magnetic fields. Combined with frequency shift-based readouts, this design enabled rapid, high-precision detection of serum cardiac biomarkers for acute myocardial infarction, advancing the clinical applicability of SERS in personalized diagnostics [122].

Overall, recent advances in plasmonic metasurfaces for SERS have driven the field toward greater sensitivity, reproducibility, and versatility. This progress has been enabled by innovations in structural design, such as three-dimensional architectures, anisotropic nanostructures, and chiral configurations, as well as the development of scalable and sustainable fabrication methods. These technological improvements have expanded the practical applicability of SERS-based platforms to diverse domains including wearable biosensors, portable environmental monitors, and multiplexed clinical diagnostics. Moreover, the integration of complementary approaches—such as reusable sensor surfaces, semiconductor-enhanced plasmonics, and AI-driven data analysis—has further accelerated the transition of SERS from laboratory research to real-world deployment. As these trends continue to evolve, plasmonic metasurfaces are poised to become foundational components of next-generation analytical tools, with broad impact across analytical chemistry, biomedical diagnostics, and environmental monitoring.

### 3.2. SEIRA

SEIRA has emerged as a powerful extension of plasmonic sensing, complementing techniques like SERS by enabling the detection of molecular vibrational fingerprints in the mid-infrared range. By coupling molecular absorption with the localized electromagnetic fields of engineered plasmonic structures, SEIRA provides label-free, highly sensitive detection of a wide range of chemical and biological species. Recent advances in metasurface design have significantly enhanced SEIRA performance, offering tailored resonance control, improved signal enhancement, and compatibility with complex analytes and environments. In what follows, we review key developments in SEIRA-enabled plasmonic metasurfaces.

**Figure 9:**
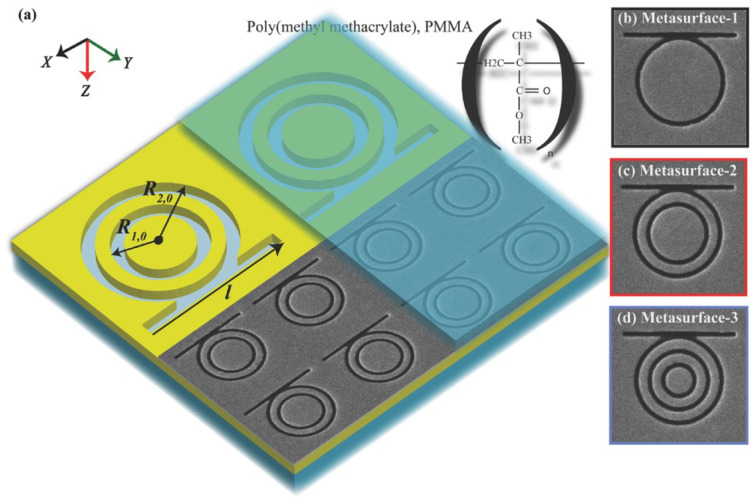
(**a**) Schematic diagram of the proposed metasurface consisting of concentric annular and rectangular apertures. The specific geometrical parameters of the unit cell are $R_{2,0}$ = 1 µm, $R_{1,0}$ = 0.8 µm, and l = 2.5 µm. (**b**–**d**) The SEM images of the unit cell of the fabricated samples are shown. A 50 nm thin layer of poly(methyl methacrylate), PMMA, is spin-coated on top of the metasurface. Reprinted with permission from [123]. © 2016 WILEY-VCH Verlag GmbH & Co. KGaA, Weinheim.

A noteworthy demonstration by Dayal et al. introduced high-Q Fano-resonant metasurfaces for multiband SEIRA, using concentric annular and rectangular apertures milled into a gold film. Their design enabled the formation of hybrid plasmon–phonon modes through polarization-controlled coupling of super-radiant and sub-radiant plasmonic resonances. This allowed spectral matching with multiple vibrational modes of poly(methyl methacrylate) (PMMA), resulting in strong, tunable enhancement across several absorption bands. The work showcased a versatile platform for label-free, broadband molecular sensing using tailored Fano-resonant metasurfaces (Figure 9) [123]. Same year, De Marcellis et al. introduced a systematic approach to optimize 2D arrays of gold nanoantennas with rod and cross-geometries for SEIRA enhancement. By tailoring parameters such as antenna shape, size, and array periodicity, they demonstrated over a $10^{4}$-fold increase in signal sensitivity relative to unstructured gold films. Their analysis also highlighted the superior performance of cross-shaped nanoantennas due to their polarization insensitivity and stronger near-field confinement [124]. Soon after, Di Meo et al. expanded on this concept by fabricating large-area metasurfaces composed of these cross-shaped gold nanoantennas, achieving experimental enhancement factors as high as 48,000. Their design enabled highly sensitive, label-free detection of molecular vibrational modes, such as nitrile and carbonyl groups, at concentrations as low as 0.7 femtomoles. The device also demonstrated excellent durability and reusability, reinforcing its potential for practical infrared sensing applications [125].

Expanding the functionality of SEIRA platforms, Armelles et al. proposed a novel approach to modulate SEIRA signals using spintronic metasurfaces composed of randomly placed arrays of aligned rods and slits fabricated from giant magnetoresistive (GMR) Ni_81_Fe_19_/Au multilayers. These complementary structures—rods with electric dipolar resonances and slits with magnetic dipolar resonances—exhibited strong magnetic modulation of both far-field and near-field optical responses under an external static magnetic field. Particularly notable were the magnetically modulated near-field hot spots, where the intensity modulation of the electric or magnetic field reached values up to 1%, significantly surpassing the far-field modulation. This Magnetic Modulation SEIRA (MM-SEIRA) technique offers enhanced spatial and spectral control of molecular vibrational signals, establishing a promising route for highly sensitive, contactless IR sensing platforms [126].

In a parallel effort to enhance spectral versatility, Zvagelsky et al. demonstrated the use of gold Y-shaped nanoantennas as SEIRA substrates for probing thin films of tris(8-hydroxyquinoline) aluminium (Alq_3_), a key material in organic optoelectronics. Their metasurface design supported two independent plasmonic resonances, enabling simultaneous enhancement of multiple vibrational modes within the mid-IR fingerprint region. SEIRA measurements revealed a clear dependence of signal intensity on film thickness, with a saturation effect occurring at thicknesses approaching the height of the antennas. This geometry enabled highly sensitive and polarization-dependent molecular detection, underscoring the potential of tailored metasurfaces for characterising nanoscale organic layers in multilayer optoelectronic devices [127].

An alternative strategy for integrating SEIRA metasurfaces was proposed by Vasić et al. who theoretically designed hollow metal–insulator–metal (MIM) metasurfaces. These structures consist of two metallic layers separated by a nanofluidic channel that facilitates direct infiltration of liquid analytes into the regions of maximal electric field enhancement. This architecture improves the spatial overlap between the analyte and the localized fields, thereby boosting sensitivity. The metasurface operates in an overcoupled regime—where radiative losses dominate over non-radiative decay—achieving an absorption sensitivity exceeding 10 RIU^−1^, which enables detection of variations in the imaginary part of the refractive index below $10^{-3}$. It is important to note that this study was purely theoretical, combining temporal coupled mode theory and numerical simulations to establish optimal design principles [128].

Meanwhile, Zhang et al. (2023) [129] reported a broadband, multi-resonant infrared metasurface sensor for label-free detection of breast cancer-associated mirna biomarkers. Their experimental platform is based on a multi-well SEIRA sensor chip fabricated with periodic arrays of gold nanorods on a CaF_2_ substrate. Each nanorod pair within a unit cell is designed to produce two distinct resonant modes—covering the spectral fingerprint regions of 800–2000 cm^−1^ and 2800–3500 cm^−1^—to maximize overlap with the vibrational absorption bands of target miRNAs. This metasurface structure achieves a simulated SEIRA enhancement factor of up to $10^{3}$. The fabricated nanostructures were experimentally validated and integrated with an FTIR microscope system, enabling multi-target and multiplexed detection. The system’s performance demonstrated excellent agreement with standard molecular assays such as qPCR and next-generation sequencing (NGS), underscoring its viability for rapid and sensitive early cancer diagnostics. In a related effort, Huang et al. (2023) [130] demonstrated the versatility of plasmonic metasurfaces by integrating them into multiwell cell-culture chambers for live-cell assays. Their platform facilitated real-time measurements of cell adhesion and drug responses, confirming the compatibility of SEIRA with dynamic biological environments.

**Figure 10:**
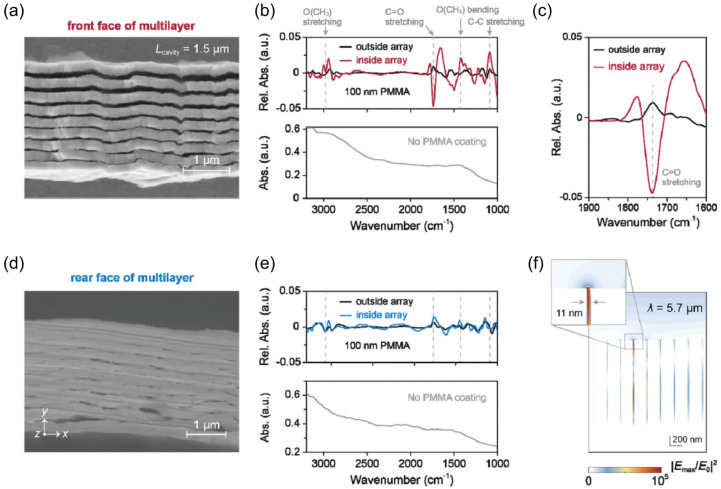
Ultrasensitive chemical fingerprinting via nanocavity-enhanced absorption in FP-dPNA structures. (**a**) SEM of the front face of FP-dPNA multilayer with 1.5 µm cavity post-sectioning. (**b**) Relative absorption spectra inside (red) and outside (black) nanocavities with 100 nm PMMA coating (top); pristine sample inside cavities (bottom). Gray dashed lines indicate PMMA vibrational bands. (**c**) Zoomed view of the C=O vibrational band of PMMA. (**d**) SEM of rear face of multilayer (1.5 µm cavity). (**e**) Absorption spectra inside (blue) and outside (black) PMMA-coated nanocavities on the rear face. (**f**) Simulated electric field intensity at 1734 cm^−1^, showing resonance in an 11 nm wide nanocavity. Reprinted with permission from [132]. © 2023 by Wiley-VCH GmbH.

Dixon et al. introduced an innovative broadband SEIRA platform based on dispersion-engineered plasmonic Fabry–Pérot (FP) nanocavity arrays (FP-dPNAs), achieving near-field enhancements up to $10^{6}$, two to three orders of magnitude higher than conventional metasurface systems. These metasurfaces utilize MIM cavity arrays with gradually tapered widths, enabling subwavelength confinement and selective resonance over a broad spectral range in the mid-IR. The design allows resonant enhancement across multiple vibrational modes on a single device, addressing a critical limitation of traditional narrowband SEIRA substrates. Importantly, the FP-dPNA structures were fabricated via a lithography-free nanoskiving technique, offering high-throughput, scalable production of ultra-narrow nanocavities with aspect ratios up to 500. The platform demonstrated highly sensitive label-free detection of thin PMMA films, resolving multiple vibrational signatures across the IR spectrum, with a reported enhancement factor of ≈$8\cdot 10^{4}$ at the C=O stretching band (1734 cm^−1^). Figure 10 highlights this performance, showcasing detailed SEM images of the nanocavity architecture together with the enhanced absorption spectra resulting from plasmonic resonance.

Finally, Dang et al. (2024) [131] presented a dual-band plasmonic metasurface design based on SLRs in the mid-infrared range, achieved by combining two types of square arrays of gold microdots with different dimensions. This configuration enabled simultaneous enhancement of methyl and amide vibrational bands, facilitating label-free, real-time monitoring of biomolecular interactions. Notably, the diagonally symmetric structure eliminated the need for in-plane polarization control of the incident infrared light, thereby improving detection robustness and operational simplicity. Their study underscores the potential of dual-resonance SEIRA platforms in high-sensitivity, on-site biosensing applications.

Taken together, these advancements underscore the transformative potential of engineered plasmonic metasurfaces in SEIRA-based sensing. As highlighted in Table 1, a diverse array of metasurface designs—from high-Q Fano resonances in concentric ring apertures [123] to dual-resonant Au microdot arrays [131]—have been developed to overcome longstanding challenges in mid-infrared molecular detection. By leveraging precise geometric tailoring (cross- and Y-shaped nanoantennas, rod–slit combinations, or ultranarrow Fabry–Pérot-type nanocavities), researchers have achieved exceptional sensitivity and spectral tunability, with enhancement factors ranging from approximately $10^{3}$ to $10^{6}$. This level of performance not only enables reliable detection of trace analytes such as low-concentration molecular groups [125], thin organic layers [127], and biomolecular markers [129,131] but also paves the way for real-time, in situ characterization of living cells [130].

A key driver of progress lies in the integration of novel materials and device concepts. For instance, magnetic field modulation [126] and nanofluidic channels [128] illustrate how complementary physical mechanisms and fluidic infiltration strategies can amplify SEIRA signals, enabling contactless and dynamically reconfigurable sensing. Moreover, approaches such as lithography-free nanoskiving for subwavelength cavity fabrication [132] highlight the push toward more scalable, cost-effective production methods suitable for broad deployment. Equally important is the trend toward broadband and multi-resonant operation: by supporting multiple resonances within one device, metasurfaces can match distinct vibrational signatures across the mid-IR spectrum, enhancing both diagnostic accuracy and sample throughput.

In sum, these innovations converge toward a shared vision of robust, high-throughput, and highly sensitive SEIRA platforms capable of detecting a wide range of chemical and biological targets. As fabrication techniques evolve and metasurface architectures become increasingly sophisticated, SEIRA is positioned to complement and extend traditional infrared spectroscopy methods. The field’s rapid development, evidenced by the substantial enhancement factors and ever-broadening range of analytes summarized in Table 1, reinforces the view of SEIRA-enabled metasurfaces as a powerful analytical tool with the potential for transformative impact in chemistry, life sciences, and clinical diagnostics alike.

### 3.3. SEF

To conclude this section on plasmonic-type metasurfaces, we now turn to their application in SEF. This powerful technique has also garnered significant attention for its ability to boost the sensitivity of optical detection in biosensing and spectroscopy. As with other signal enhancement methods, plasmonic metasurfaces, with their ability to confine electromagnetic fields and support high-emissivity environments, play a central role in amplifying fluorescence signals and advancing SEF performance.

Choi et al. (2015) presented a strategy to mitigate metal-induced fluorescence quenching by incorporating subnanometer-thick self-assembled monolayers (SAMs) as dielectric spacers between dye molecules and metallic nanostructures [133]. Their study demonstrated substantial suppression of quenching and a remarkable increase in fluorescence enhancement on stacked complementary plasmo-photonic metasurfaces, which support hybrid resonances combining plasmonic and photonic crystal modes. Using an ultrathin SAM, they achieved average enhancement factors (EFs) exceeding 2600-fold for Rhodamine 590, relative to a Si wafer reference. This enhancement was attributed to a synergistic combination of high emittance, reduced nonradiative decay pathways, and strong but uniform electric-field confinement, minimising reliance on localized “hot spots” and enabling consistent enhancement across the illumination area.

**Figure 11:**
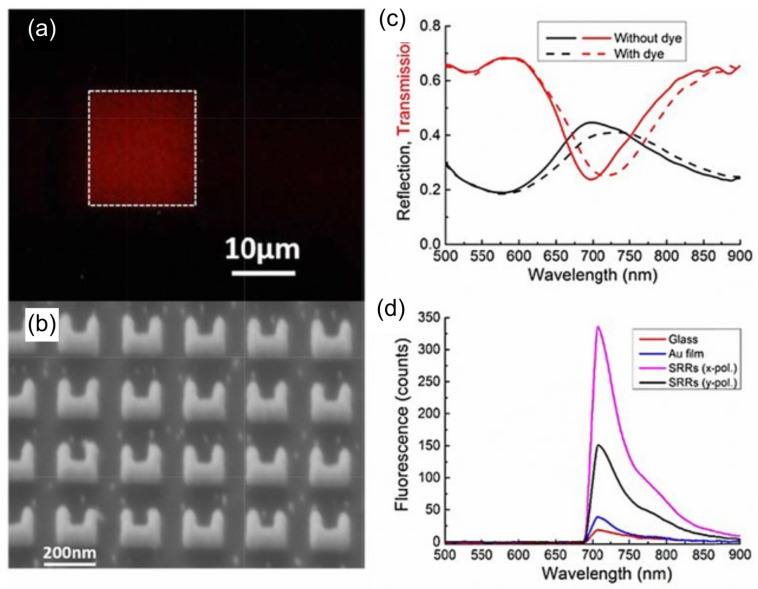
Fluorescence properties of the SRR-based metasurface designed by Luo et al. (**a**) Microscopy image of the fluorescence emission from the sample under excitation by a 671 nm laser at an oblique incidence of 77°. The boundaries of the SRR-based metasurface are marked with white dotted lines. (**b**) SEM image showing a section of the fabricated SRR-based metasurface from (**a**). (**c**) Transmission and reflection spectra of the SRR-based metasurface measured under white light illumination, both without (solid lines) and with (dashed lines) a dyed PVA film coating. (**d**) Enhanced fluorescence spectra of the SRR-based metasurface coated with a dyed PVA film under x-polarized (pink) and y-polarized (black) excitation. For reference, fluorescence spectra of dyed PVA films on gold (blue) and glass (red) substrates are also included. Reprinted with permission from [134]. © 2017 by WILEY-VCH Verlag GmbH & Co. KGaA, Weinheim.

Shortly after, Luo et al. (2017) demonstrated that split-ring resonator (SRR)-based plasmonic metasurfaces can effectively control fluorescence via engineered electric and magnetic modes [134]. Under oblique incidence (77°), x-polarized excitation activated a magnetic mode that enhanced Rhodamine 800 fluorescence by 18 fold, maintaining the emission polarization. In contrast, y-polarized excitation triggered a different magnetic mode, only excitable at oblique angles, resulting in an 8-fold enhancement and rotated emission polarization due to the interplay of electric and magnetic resonances. As shown in Figure 11, fluorescence from the SRR metasurface significantly exceeded that of flat gold or glass references, confirming the strong modal coupling and polarization-dependent control.

Another significant development was reported by Qin et al. (2017), who introduced ultrathin optical magnetic mirror metasurfaces composed of periodic nanogaps that confined the electric field to a ≈15 nm region, delivering fluorescence enhancements as high as 45-fold [135]. These metasurfaces rely on nano-groove arrays on a single-crystalline gold microplate, with subwavelength period and depth. By concentrating strong electric fields in a deep subwavelength region close to the metal surface, fluorescent materials deposited on the mirror exhibited substantially increased emission intensity. Further modulation of enhancement was achieved by varying the incident polarization, underscoring the versatility of ultrathin magnetic mirrors in fluorescence amplification.

Subsequent innovations include the work of Narasimhan et al. (2019), who developed biomimetic gold metasurfaces using a scalable three-step fabrication process to create metal–insulator–metal (MIM) architectures with 5 nm insulating nanogaps [136]. These structures, comprising closely coupled nanodisks and nanoholes, support quadrupolar gap-plasmon modes in the visible to near-infrared range, effectively suppressing quenching while maintaining high fluorescence enhancements. The accessible nanogaps enable functionalization for biosensing applications, allowing selective detection of biomolecules such as the streptavidin–biotin complex and nucleic acid sequences. Reported fluorescence enhancements reached up to 501-fold for different fluorophores, and the system enabled sensitive detection of single-stranded DNA, including HIV-1 gene segments, across a concentration range from 10 pM to 10 µM. Notably, a 5.4-fold fluorescence increase was observed in cell lysate assays targeting CD4 mRNA, demonstrating the platform’s promise for multiplexed and quantitative biosensing.

More recently, Iwanaga et al. (2021) showcased plasmon–photon-hybrid metasurfaces comprising perforated silicon waveguides integrated with stacked complementary gold nanostructures [137]. These high-emittance metasurfaces were fabricated using electron beam lithography on silicon-on-insulator wafers, followed by gold deposition, and were integrated into microfluidic chips for biosensing. The design supports uniform and reproducible fluorescence enhancement, avoiding reliance solely on local hot-spots and instead leveraging engineered broadband optical resonances. Using this platform, direct and indirect fluorescence assays demonstrated sensitive detection of immunoglobulin G (IgG) with a detection limit of 34 fM, anti-p53 antibodies at 50 pg mL^−1^, and complementary DNA strands of SARS-CoV-2 RNA at femtomolar concentrations. These results highlight the practical versatility of hybrid metasurfaces for detecting diverse biomarkers with high sensitivity and reliability.

In a cost-effective alternative approach, Anăstăsoaie et al. (2022) developed random plasmonic metasurfaces by thermally annealing thin metallic films deposited on glass and silicon substrates [138]. These metasurfaces consist of randomly distributed aggregates of metallic nanoparticles (Au, Ag, or Al) that support plasmonic resonances, intensifying local electromagnetic fields and enhancing fluorescence emission. Finite-difference time-domain simulations and experimental measurements confirmed significant field enhancement at both the excitation and emission wavelengths of Rhodamine 6G. Fluorescence enhancements up to 423-fold were observed, depending on metal type and nanostructure morphology. While lacking the precise field control of engineered metasurfaces, the simplicity, scalability, and high enhancement factors of this method make it highly promising for large-area, low-cost fluorescence biosensing platforms.

In Table 2, these metasurfaces are summarized along with their respective characteristics. Together, the advancements illustrate a rapidly evolving landscape in plasmonic metasurface design for fluorescence enhancement, with strategies ranging from nanospacers and tunable resonant modes to random nanoparticle arrays. Ongoing research integrating next-generation metasurface architectures with biofunctionalization protocols and advanced optical detection schemes promises to further revolutionize both optical sensing and spectroscopy.

## 4. Dielectric Metasurfaces for Enhanced Spectroscopy

Dielectric metasurfaces present several notable advantages over their plasmonic counterparts, including reduced optical losses, higher resilience to laser-induced damage, and the capacity to excite both electric and magnetic resonances [34]. Such features mitigate the detrimental heating effects commonly encountered in metal-based designs and allow for robust field enhancement via Mie-type resonances. Consequently, dielectric metasurfaces are particularly well suited to applications where high sensitivity and reproducibility are essential.

In the following subsections, in a similar manner to the plasmonic metasurfaces section, we explore recent advancements in dielectric metasurfaces for SERS, SEIRA, and SEF. We discuss how the intrinsic properties of these materials contribute to the performance and applicability of these techniques and highlight the latest developments and challenges in the field, underscoring the future potential of dielectric metasurface-based sensing platforms.

### 4.1. SERS

For SERS, dielectric metasurfaces have recently emerged as a promising alternative to traditional metallic platforms. These materials leverage their ability to support robust dielectric resonances, enabling strong electromagnetic field confinement without the associated optical losses characteristic of plasmonic metals. Moreover, dielectric metasurfaces offer tunable optical properties, low absorption, and compatibility with CMOS fabrication technologies, opening avenues for scalable, efficient, and biocompatible SERS platforms for a variety of sensing applications.

**Figure 12:**
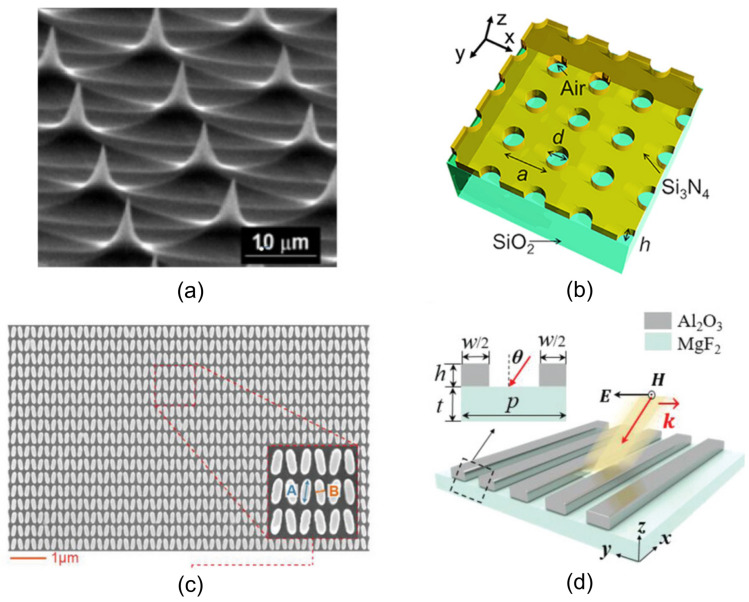
Dielectric metasurfaces designed for SERS. (**a**) Scanning electron microscopy (SEM) image of a silicon tip-shaped metasurface. Reprinted with permission from [139], © 2017 Optical Society of America. (**b**) Schematic layout of a two-dimensional square photonic crystal metasurface (PhCM), designed and fabricated by Romano et al. Reprinted with permission from [140]. © 2018, American Chemical Society. (**c**) SEM image of an experimental metasurface featuring a dual-ellipse geometry. Reprinted from [141]. Licensed under CC BY 4.0. (**d**) Schematic illustration of the all-dielectric SERS metasurface with Al_2_O_3_ and MgF_2_ layers arranged in a dielectric metagrating, where the period p=475 nm, thickness t=100 nm, and height h=110 nm. Reprinted with permission from [142]. © 2023 Wiley-VCH GmbH.

Lagarkov et al. investigated a tip-shaped silicon metasurface composed of a periodic array of submicrometer silicon cones arranged on a square lattice (period ≈ 2.1 µm, tip radius <10 nm, and height 0.3–0.7 µm); see Figure 12a. Using both theoretical analyses and finite element simulations, they demonstrated that these cone structures exhibit multiple dielectric resonances capable of markedly enhancing local optical fields. Experimental measurements further confirmed anomalous optical responses arising from these resonances. Notably, decorating the cone tips with gold nanoparticles (AuNPs) created hybrid metal-dielectric resonances, boosting local electric fields by over three orders of magnitude. In certain configurations with closely spaced AuNPs, simulations predict enhancements SERS factors of 10^8^–10^11^. Consequently, Raman signals from adsorbed DTNB (5,5′-dithio-bis-[2-nitrobenzoic acid]) molecules experienced enhancements of several orders, establishing silicon-based dielectric metasurfaces as highly effective SERS platforms for sensitive chemical and biological detection [139].

Romano et al. (2018) developed an innovative all-dielectric metasurface platform composed of silicon nitride (Si_3_N_4_) (Figure 12b), optimized to support bound states in the continuum (BICs) [140]. By precisely tuning geometric parameters such as lattice periods and hole dimensions, the authors achieved strong BIC resonances in the visible range (530–560 nm), enabling high optical field concentrations without the large absorption losses typically associated with metals. These well-defined BIC modes significantly boosted Raman and fluorescence signals by factors up to 10^3^ and were further enhanced when combined with LSPRs from plasmonic nanoparticles. Overall, their approach established a low-loss dielectric platform that capitalizes on BIC physics for highly sensitive spectroscopic applications.

Continuing this line of research, Hu et al. introduced a titanium dioxide (TiO_2_)-based platform exploiting BIC resonances for enhanced SERS performance [141]. Their top-down nanofabrication approach yielded precisely patterned metasurfaces composed of hole-in-disk and paired elliptical nanostructures (Figure 12c). By carefully engineering resonant absorption characteristics, they achieved substantial electric field enhancements ($|E/E_{0}{|}^{2}\approx 10^{3}$), optimizing the photoinduced charge transfer (PICT) effects central to chemical sensing. Importantly, the flexibility in tuning BIC resonances facilitated resonance matching with specific molecular absorption wavelengths, significantly enhancing detection sensitivity. The TiO_2_ metasurfaces developed by Hu et al. effectively resolved key limitations of previous semiconductor SERS designs, achieving detection sensitivities down to 10^−8^ M, surpassing conventional metallic substrates.

Chen et al. (2024) further extended these concepts by developing an Al_2_O_3_/MgF_2_ metasurface (Figure 12d) exploiting quasi-bound states in the continuum (Q-BIC) within a strong-coupling regime [142]. This structure leveraged deep learning (transformer-based models) to optimize metasurface geometry, resulting in a significant advancement by achieving substantial SERS enhancements ($EF\approx 10^{7}$). The critical innovation lay in utilizing angle-dependent strong coupling to precisely tune the Q-BIC resonance, greatly expanding the spatial and intensity scale of electromagnetic fields compared to conventional weakly coupled dielectric and metallic substrates. Additionally, they demonstrated that these metasurfaces significantly outperform metallic alternatives, both in near-field intensity enhancement and field spatial extension, marking a significant step towards CMOS-compatible, metal-free, high-performance SERS platforms.

Table 3 summarizes the previously mentioned dielectric metasurfaces for SERS. These investigations underscore the pivotal role of bound states in the continuum, whether strictly protected or quasi-bound, in the design of next-generation dielectric metasurfaces for SERS. By finely adjusting geometrical parameters to tailor high-Q resonances, researchers have not only reduced absorption losses, but also expanded the spatial and spectral reach of electromagnetic field enhancements. This powerful combination positions BIC-driven all-dielectric metasurfaces as highly attractive for advanced sensing, imaging, and on-chip integration.

### 4.2. SEIRA

In the case of SEIRA, dielectric metasurfaces also provide advantages by overcoming some of the limitations associated with metallic nanostructures. As previously mentioned, in contrast with plasmonic metasurfaces, which typically suffer from high ohmic losses and broader resonances, high-index dielectric platforms (e.g., silicon or germanium) exhibit substantially lower intrinsic losses and can sustain high-quality-factor (high-Q) resonances with strong near-field enhancement. As we see, these attributes enable more precise detection of molecular vibrational signatures at lower analyte concentrations and facilitate on-chip integration for compact mid-infrared sensors.

**Figure 13:**
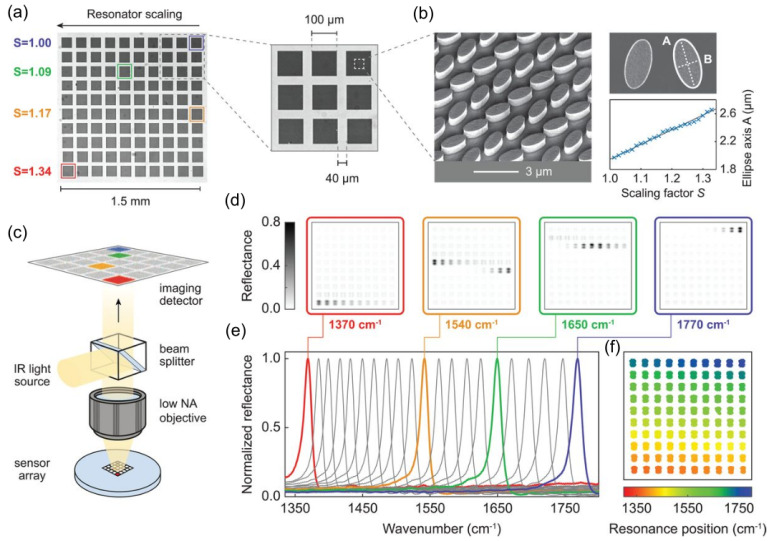
The experimental realization by Tittl et al. of the pixelated metasurface. (**a**) Optical microscopy images showing the fully fabricated pixel array, composed of 100 individually tuned elements. (**b**) SEM micrographs confirming that the dimensions of each elliptical resonator vary linearly with the scaling factor. (**c**) Schematic of the mid-infrared (mid-IR) microscopy system employed for reflectance imaging. (**d**) Reflectance maps of the metasurface recorded at four distinct wavenumbers in the mid-IR range. (**e**) Normalized reflectance spectra from 21 out of the 100 metapixels, highlighting the correspondence between colored peaks and the images in (**d**). (**f**) Extracted resonance frequencies for all metapixels across the array. Reprinted with permission from [143]. © 2018, The American Association for the Advancement of Science.

A demonstration of these capabilities was reported by Tittl et al. (2018), who introduced a pixelated dielectric metasurface based on hydrogenated amorphous silicon (a-Si:H) resonators engineered to exhibit ultrasharp resonances spanning a broad frequency range [143]. Each metapixel in the two-dimensional array is scaled to resonate at a distinct frequency, and taken together, the pixels form a “molecular barcode” that directly encodes multiple vibrational fingerprints into a spatial reflection pattern. This approach eliminates the need for bulk optical spectrometers or mechanically tunable systems. Their nanofabrication strategy, employing electron-beam lithography and reactive ion etching, produced a 10 × 10 array of anisotropic elliptic resonators with resonance frequencies tuned by adjusting the unit cell dimensions. High-Q (Q > 200) supercavity modes, rooted in bound-states-in-the-continuum physics, yielded pronounced field enhancements and sensitive detection of biological, polymer, and pesticide molecules on the metasurface. Figure 13 illustrates the core aspects of their device, including the reflective imaging approach and the tunable elliptical resonators. This mid-IR nanophotonic sensor based on all-dielectric high-Q metasurface elements demonstrates its capability to enhance, detect, and differentiate the absorption fingerprints of various molecules, exploiting the collective behavior of Mie resonances, which manifest as supercavity modes driven by the physics of bound states in the continuum.

Most recently, Richter et al. (2024) expanded these concepts by introducing gradient high-Q dielectric metasurfaces based on Ge elliptical resonators on a CaF_2_ substrate [144]. Rather than assigning discrete resonances to each pixel, they gradually varied the unit-cell geometry across one dimension of the metasurface, creating a continuous gradient of resonance frequencies. This gradient design provides wide spectral coverage without significantly increasing the device footprint and allows the sampling density to be tailored to specific molecular absorption bands of interest. Applications demonstrated by them range from monitoring polymer mixtures with overlapping absorption features to conducting multistep bioassays on a single chip. Moreover, they observed vibrational strong coupling in polymer thin films, revealing that gradient metasurfaces can serve as testbeds for fundamental light–matter interactions. By harnessing the SEIRA effect in a continuously varying resonator design, they achieved significant signal enhancements, on the order of factors of 50 or more, relative to conventional transmission measurements, illustrating the transformative potential of dielectric metasurfaces for miniaturized, high-sensitivity mid-IR spectroscopy.

These studies underscore how high-Q dielectric resonators, whether discretely pixelated or arranged in resonant gradients, can deliver broad yet finely resolved spectral coverage in a form factor compatible with scalable, low-cost fabrication. The Tittl design demonstrates the effectiveness of spatially encoded molecular barcodes, while Richter extends the approach to gradient-resonance devices that elucidate novel physical phenomena. Together, these advances pave the way for next-generation mid-IR sensors that unite chemical specificity with the practical advantages of chip-scale photonic integration.

### 4.3. SEF

To finish this section on dielectric metasurfaces, we now turn to their application in SEF. Several research efforts have demonstrated the effectiveness of dielectric metasurfaces in enhancing fluorescence intensity and sensitivity. Iwanaga et al. (2018) demonstrated impressive fluorescence intensity enhancement by employing silicon rod array metasurfaces designed with resonances matched precisely to fluorescent dye absorption spectra [145]. By achieving fluorescence enhancements exceeding 1000-fold compared to flat silicon substrates, their work highlighted the crucial role of guided modes and higher-order magnetic resonances. Such outstanding performance places dielectric metasurfaces on par with the most effective metallic-based platforms traditionally used for fluorescence enhancement, suggesting their potential for broad biomolecular sensing applications.

Expanding the potential of dielectric metasurfaces beyond mere intensity enhancement, Lee et al. (2019) explored their capability in structured illumination microscopy [146]. Through all-dielectric metasurfaces based on hydrogenated amorphous silicon (a-Si:H), encoded illumination patterns were generated, significantly improving lateral imaging resolution. Their metasurfaces produced structured illumination without the need for high-power lasers, making this approach more accessible for practical biomedical imaging. Experimentally, they demonstrated a notable improvement of 1.71-fold in resolution, effectively overcoming conventional diffraction limitations. Although fluorescence intensity enhancement was not reported, the increased spatial resolution allows resolving more emitters per area, enhancing the quantity and quality of extractable spectroscopic information. Instead of near-field amplification, the metasurface improves analysis via super-resolved structured illumination. This innovative use underscores the flexibility and potential of dielectric metasurfaces as multifunctional optical devices, expanding their application into holography, computational imaging, and encryption technologies. Further building upon dielectric metasurfaces’ unique optical characteristics, Solomon et al. (2020) demonstrated their use in enhancing fluorescence-detected circular dichroism (FDCD) [147]. In their studies, silicon nanodisk arrays were engineered to exhibit overlapping electric and magnetic dipolar Mie resonances, carefully aligned with molecular circular dichroism signals. This precise spectral overlap significantly increased the optical chirality density, amplifying differential absorption of chiral molecular monolayers. This technique effectively distinguished different molecular conformations, exemplified by DNA hybridization and de-hybridization processes. Such enhancements of intrinsic molecular circular dichroism pave the way for potential applications in enantioselective photolysis and sensitive molecular diagnostics.

There have also been theoretical developments in nanophotonic platforms tailored for quantum emitters and anisotropic metasurfaces. Fang et al. investigated resonance fluorescence properties using cascaded exciton–biexciton quantum dots coupled to two-dimensional black phosphorus (BP) metasurfaces [148]. They demonstrated through simulations that tunable elliptically polarized surface plasmon modes can be achieved by varying carrier concentration, significantly enhancing spontaneous decay and enabling quantum information applications. Their theoretical work also revealed the potential of gate doping to enhance two-mode squeezing in resonance fluorescence. Liu et al. (2023) experimentally demonstrated the efficacy of dielectric metasurfaces by employing silicon metasurfaces sustaining symmetry-protected BICs to enhance the near-infrared emission of PbS colloidal quantum dots (CQDs) [149]. They observed a fluorescence enhancement factor of approximately 10-fold with a high-quality factor of 251 at wavelength 1408 nm, highlighting its suitability for on-chip silicon-based optical sources and integrated sensors.

**Figure 14:**
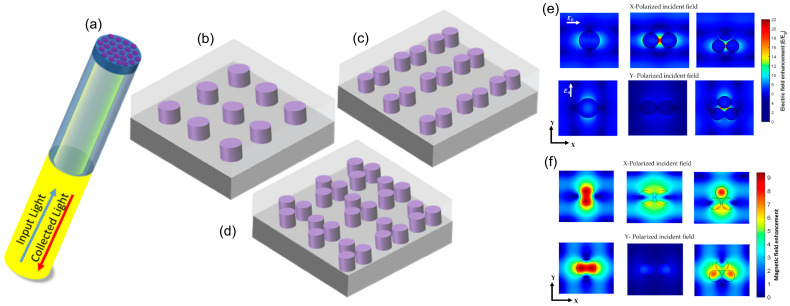
Schematic representation of the geometries studied by Alahmby et al. (**a**) Diagram of a fibre with NSs fabricated at its tip. Square-periodic arrays of (**b**) cylindrical Si NSs, (**c**) Si cylindrical dimer NSs, and (**d**) Si cylindrical trimer NSs on an SiO_2_ substrate, all immersed in a homogeneous medium with a refractive index of 1.33. Simulated field maps showing the enhancement of (**e**) electric and (**f**) magnetic intensity distributions in the plane located at the center of the nanostructures. The incident field is excited at λ = 650 nm. Reprinted from [150]. Licensed under CC BY 4.0.

Another significant advancement, presented by Alhalaby et al. (2022) through simulations, explored the integration of fluorescence-enhancing dielectric metasurfaces onto multimode optical fibres for optrode-based biosensing applications [150]. They utilized arrays of cylindrical silicon nanoantennas arranged as single units, dimers, and trimers. Their simulated metasurface configurations predicted fluorescence enhancements of up to three orders of magnitude. Notably, dimer structures provided the highest enhancement due to enhanced resonant-driven excitation in gaps. This theoretical study additionally considered realistic biosensing scenarios, accounting for the random orientation and distribution of emitters, proving metasurface–fiber integration feasible and highly promising for clinical sensing devices. Figure 14 illustrates the different metasurface configurations evaluated, along with the simulated electric and magnetic field distributions under plane-wave excitation.

Finally, Zhai et al. (2024) approached the challenge of photoluminescence (PL) extraction efficiency from dye films through a novel integration of dye layers opposite c-Si metasurfaces on quartz substrates [151]. This approach, distinct from traditional spin-coating methods, significantly improved fluorescence output by efficiently redirecting trapped photons via metasurface diffraction, achieving a 2.5-fold enhancement in PL. Their approach highlighted how dielectric metasurfaces could overcome internal reflection losses, facilitating more efficient, uniform emission in luminescent devices, further extending the potential practical applications of metasurface-based fluorescent devices across the visible spectrum.

## 5. Discussion

Surface-enhanced spectroscopy (SERS, SEIRA, and SEF) originated from curious observations in the 1970s, when researchers discovered that molecules on roughened metal surfaces, particularly silver, exhibited unexpectedly strong Raman signals. Early experiments by Fleischmann and colleagues laid the groundwork for SERS, later elaborated by Jeanmaire, Van Duyne, and Creighton, who demonstrated that weak Raman scattering could be amplified by several orders of magnitude through LSPs on nanostructured metals. At around the same time, reports of SEIRA showed that molecules deposited on thin metal films displayed significantly increased IR absorption, addressing a similar sensitivity issue that hampered conventional IR spectroscopy. Fluorescent enhancement was also hypothesised in the 1970s, with Drexhage showing that metal proximity could alter emission intensity and lifetime. However, those early studies often observed quenching, so practical SEF did not truly flourish until the 1990s, aided by plasmonic nanoparticles and spacer layers that controlled molecule–metal distances.

Today, each technique addresses different spectroscopic challenges: SERS enhances the inherently weak Raman signal, SEIRA amplifies IR absorption tied to dipole changes, and SEF increases fluorescence brightness while mitigating quenching. Though all rely on coupling light to materials at the sub-wavelength scale, they provide distinct types of information, from the vibrational “fingerprint” of a molecule (SERS) or IR-active bonds (SEIRA) to the labelled or intrinsic fluorescence output (SEF).

Specifically, in SERS, incident light excites resonant modes, creating intense electromagnetic field enhancements that amplify Raman scattering by orders of magnitude, enabling detection of minute analyte quantities—even single molecules. This high sensitivity comes with challenges in reproducibility, as only molecules very close to these field enhancements show strong signals, making uniform coverage essential. SERS spectra offer a detailed vibrational “fingerprint,” identifying molecular structures and orientations based on which Raman modes are enhanced.

By contrast, SEIRA leverages enhanced electric fields in the infrared, which highlight vibrational modes involving changes in dipole moment. Polar bonds like C=O or O–H, typically weak in Raman, often dominate IR spectra, so SEIRA delivers complementary information. Although IR peaks can be broader than Raman features, SEIRA’s signal scales more directly with concentration, allowing simpler quantitative analysis.

SEF extends the utility of fluorescence, which is intrinsically sensitive but often limited by low emission or photobleaching. By placing fluorophores near metals at an optimal distance, SEF can amplify emission, reduce required excitation power, and enhance signal-to-noise ratios. Unlike SERS or SEIRA, SEF usually lacks rich structural details because fluorescence spectra are broad, but it excels in imaging and high-throughput assays, common in biological research.

Hence, while SERS and SEIRA provide unambiguous molecular fingerprints, SEF offers enhanced brightness and convenience, making all three methods valuable, depending on whether molecular identification, functional group analysis, or ultrasensitive fluorescence detection is the priority.

The advent of metasurfaces and advanced nanostructures has significantly expanded the possibilities for surface-enhanced spectroscopy, allowing unprecedented control over optical fields at the nanoscale. In early SERS and SEIRA research, substrates were often random or semi-controlled, relying on chemically roughened metals or aggregated colloids. By contrast, metasurfaces use carefully engineered arrays of resonant elements, such as bowtie antennas or split-ring resonators, to concentrate electromagnetic fields at precise locations. This approach increases enhancement factors and, crucially, improves reproducibility, since each element can be nearly identical in shape, size, and spacing. For SERS, metasurfaces enable more quantitative measurements, as the uniform hot-spots can provide consistent signals across large substrate areas. In SEIRA, patterned nanostructures can be designed to function as strong absorbers at specific IR frequencies, significantly boosting sensitivity to molecules that exhibit characteristic vibrational bands. Furthermore, these designs can be tuned simply by altering geometric parameters, tailoring the resonances to match a desired molecule’s IR signature. Metasurfaces can also be integrated onto various platforms, from silicon chips to flexible polymers, facilitating applications in remote sensing, microfluidic assays, or implantable biosensors. In SEF, precise control of nanogaps and spacer layers helps avoid quenching while maximizing fluorescence output, enabling brighter signals with lower excitation power. The ability to include multiple resonances on a single metasurface paves the way for multifunctional devices that simultaneously support SERS, SEIRA, and SEF measurements, or incorporate distinct sensing regions for multiplexed detection.

Another emerging frontier is vibrational strong coupling (VSC) [152], where a molecular vibration coherently hybridises with a confined photonic mode to form vibro-polaritons. Metasurface-based Fabry–Pérot cavities or high-Q quasi-BIC resonators can already reach Rabi splittings exceeding 100 cm^−1^ for monolayer films, signalling entry into the strong-coupling regime. Embedding SERS or SEIRA read-outs inside such cavities offers qualitatively new opportunities: mode-selective enhancement that depends not only on local field intensity but also on polaritonic character, altered line-widths and selection rules, and even modified chemical reactivity. Realizing high enhancement and strong coupling simultaneously requires careful optimization of cavity Q-factor, loss, and mode volume, all parameters that lithographic metasurfaces can tune with nanometer precision. Systematic exploration of how weak- and strong-coupling limits influence enhancement factors, spectra, and sensing figures of merit therefore represents a fertile research direction that could reshape surface-enhanced spectroscopy. By uniting nanofabrication precision with macroscale device integration, metasurfaces address many long-standing issues in surface-enhanced spectroscopy and foster next-generation sensors.

Despite these advancements, surface-enhanced spectroscopy still faces challenges that motivate ongoing research. Reproducibility is a prime concern, especially for SERS, where colossal enhancements often arise from only a few localized hot-spots. Minor variations in nanoparticle arrangement or roughness can produce large signal discrepancies. To address this, scientists employ advanced fabrication methods like nanoimprint lithography or DNA-origami assembly to create uniform nanoclusters with predictable gap sizes. Standardization efforts also include establishing reference molecules and protocols to measure enhancement factors consistently. Another obstacle is spectral overlap. Complex samples can produce many vibrational or fluorescence signals, sometimes masked by broad backgrounds or interfering peaks. Multivariate analysis and machine learning are increasingly used to untangle overlapping features and identify multiple components. In SEIRA, broad IR peaks may coincide, and specialized metasurfaces can be designed to selectively amplify specific frequencies, effectively filtering out irrelevant signals. Fluorescence-based methods face similar overlap when multiple dyes have overlapping emission spectra, but time-resolved approaches can help differentiate them by exploiting distinct lifetimes. Quenching and distance sensitivity also pose challenges, particularly for SEF, where fluorophores must be kept at just the right distance from metal surfaces. Shell-isolated nanoparticles or rigid spacers have mitigated this while also preventing chemical side reactions. For future VSC-enabled devices, coupling strength competes with metal losses, so new low-loss materials (e.g., TiN, heavily doped semiconductors) and hybrid metal–dielectric cavities may be required to maintain both high-Q and large field confinement. These technical refinements ensure that only target molecules in close proximity are enhanced, aiding biosensing where antibody- or aptamer-functionalized surfaces selectively capture analytes. Addressing these challenges not only yields more robust measurements but also fosters broader adoption, as labs can trust the reproducibility, specificity, and clarity of surface-enhanced results for real-world applications.

To summarize the practical differences between the two dominant metasurface classes, Table 4 contrasts plasmonic and dielectric platforms across the aspects most frequently discussed above. This side-by-side view highlights why plasmonic designs still dominate extreme-field applications, whereas dielectric designs excel when low loss, broad tunability, and full chip-level integration are required.

In the push toward next-generation platforms, hybrid materials and integrated devices are reshaping how SERS, SEIRA, and SEF are deployed. On one hand, in the case of hybrid materials several examples have been proposed. For example, triangular hybrid Au–polymer octupolar nanocavities have achieved SERS enhancement factors of about $9\times 10^{7}$ in the specific detection of Shiga toxins [113]. Combining metals with graphene or other 2D materials can damp unwanted fluorescence, stabilize adsorbates, and add tunable plasmonic effects in mid-IR regions. It has been demonstrated that stacked plasmo-photonic systems with self-assembled monolayers achieve $>2.6\times 10^{3}$ fluorescence enhancement for Rhodamine 590, while anisotropic grooves allow directional SERS in graphene-based systems [108,133].

On the other hand, integration is another major trend, with on-chip lasers, photodiodes, or camera systems merging with nanostructured substrates to form self-contained sensors for use in portable or point-of-care diagnostics. Microfluidic channels can direct samples over metasurface arrays, concentrating analytes at hot spots and enabling continuous monitoring in real time. A notable demonstration is the perforated silicon waveguide/gold metasurface chip of Iwanaga et al. [137], which within a microfluidic cartridge detects IgG down to 34 fM, anti-p53 antibodies at 50 pg mL^−1^, and still maintains <5 % chip-to-chip variability. Reconfigurable substrates, including nanosphere arrays and double-Fano dimers, offer reversible tuning with enhancements up to $10^{4}$, though thermal crosstalk and stability remain concerns [109,110].

In parallel, advanced data processing—often bolstered by machine learning—facilitates rapid and accurate spectral analysis, even in complex mixtures; for example, a deep-learning plasmonic–nanocube platform has already performed simultaneous single-molecule identification of three organophosphorus pesticides in river water [100].

As these techniques mature, each finds a sweet spot: SERS stands out for its single-molecule detection and detailed vibrational fingerprints, SEIRA captures the IR signatures of dipolar bonds and can operate in situ on catalytic or electrochemical interfaces, and SEF provides bright fluorescence signals for imaging and multiplexed assays. Metasurfaces unify them, offering custom-tailored resonances, higher reliability, and the ability to incorporate protective layers or functional coatings for biomedical applications. Expanding the toolkit to encompass vibrational strong coupling will add a new dimension, literally hybridising photons and phonons, to the already rich palette of surface-enhanced spectroscopies. Though challenges persist—like ensuring substrate reproducibility and disentangling overlapping signals—ongoing innovations are mitigating these issues and bringing surface-enhanced spectroscopy ever closer to wide-scale adoption. Ultimately, the synergy of nanotechnology, materials science, and spectroscopy is poised to transform SERS, SEIRA, and SEF from specialized research tools into indispensable instruments across multiple disciplines.

## 6. Conclusions

Surface-enhanced spectroscopy techniques—SERS, SEIRA, and SEF—have seen substantial refinement since their early demonstrations, becoming widely used analytical tools for molecular detection and characterization. Despite different underlying mechanisms and information content, these techniques share the same fundamental concept that carefully engineered surfaces concentrate electromagnetic fields, enabling sensitive measurements that are difficult to achieve with conventional optical methods.

In particular, recent progress in plasmonic and dielectric metasurfaces has improved and diversified the performance of surface-enhanced spectroscopy. Compared with early-generation random or semi-controlled substrates, modern metasurfaces offer a high degree of control over resonance design, spatial uniformity, and the achievable near-field enhancement. By tuning nanostructure dimensions, shapes, and materials, plasmonic metasurfaces can produce reproducible hot spots, while dielectric metasurfaces reduce optical losses, improve integrability, and introduce new design possibilities through Mie resonances. Metasurface engineering—often combining metal and dielectric components—has enabled multiwavelength platforms, miniaturized sensing chips, and hybrid devices that extend surface-enhanced spectroscopy into enhanced regimes of sensitivity and reliability.

Recent studies show that plasmonic metasurfaces give higher enhancement, while dielectric designs offer wider bandwidth and lower losses. Likewise, metal-based SEF provides strong fluorescence boosts, and dielectric methods narrow the performance gap by reducing quenching. To translate these advances into practical devices, it is essential to ensure reproducible hotspots at large scale, balance precision and cost in fabrication methods, and manage the expenses associated with advanced manufacturing. Addressing these aspects will pave the way for scalable production and broader adoption of surface-enhanced spectroscopy platforms.

Looking ahead, the convergence of advanced nanofabrication, materials science, and computational design is expected to offer further capabilities and wider adoption of metasurface-based sensors. Combining plasmonic and dielectric metasurfaces, integrating them with microfluidics, and leveraging reconfigurable components (e.g., phase-change materials, actively tunable substrates) should expand the range of possible devices. Advances in machine learning and data analytics for substrate design and signal processing will also facilitate the deployment of more compact, field-ready instrumentation. As a result, SERS, SEIRA, and SEF—each enhanced by a new generation of metasurface engineering—are likely to become important tools in biomedical diagnostics, environmental monitoring, and industrial quality control, reinforcing their role as core spectroscopic methods for future high-sensitivity chemical analysis.

## Figures and Tables

**Table 1:** Summary of plasmonic metasurfaces, sample types, and the enhancement factor (EF) for SEIRA-based sensors (PMMA = poly(methyl methacrylate); EF = enhancement factor; Alq_3_ = Tris(8-hydroxyquinolinato)aluminum(III); MIM = metal–insulator–metal; miRNA = microRNA; IgG = Immunoglobulin G).

Metasurface	Sample	Enhancement	Ref.
Concentric rings apertures with rectangular aperture	PMMA	–	[123]
Au cross-shaped nanoantennas	PMMA	EF: $4.8\times 10^{4}$	[124,125]
Randomly placed aligned rods and slits	–	3 rods, 7500 slits	[126]
Au Y-shaped nanoantennas	Alq_3_ thin films	SE: 6.2	[127]
Hollow MIM stack with fluidic channel	–	–	[128]
Short/narrow and long/wide nanorods	Breast cancer miRNA	EF: $10^{3}$	[129]
Multiwell with Au nanoantennas	Live cells	–	[130]
Fabry–Pérot-type nanocavity arrays	Ultrathin PMMA	EF: $8\times 10^{4}$	[132]
Dual square arrays with Au microdots	Protein A–IgG	EF: 383	[131]

**Table 2:** Summary of unit cell designs, detected molecules, and enhancement factors (EFs) for SEF using plasmonic metasurfaces (SC = stacked complementary; PlasPh = plasmo-photonic; SAM = self-assembled monolayer; PVA = poly(vinyl alcohol); PMMA = poly(methyl methacrylate); IgG Ab = Immunoglobulin G antibody; cDNA = complementary DNA; NPs = nanoparticles).

Metasurface	Detected Molecules	Enhancement	Ref.
SC PlasPh with coated by SAM	Rhodamine 590	>2600	[133]
Au split-ring resonator	Rhodamine 800 in PVA film	18 (X-pol), 8 (Y-pol)	[134]
Grooved Au magnetic mirror	ATTO 633 in 15 nm PMMA layer	45	[135]
Au nanodisks on nanoholes (5 nm SiO_2_ gap)	Alexa Fluor 555, 647, 750, 790	91–501	[136]
Perforated Si waveguides + SC Au NSs	IgG Ab, anti-p53 Ab, cDNA SARS-CoV-2 RNA	–	[137]
Random Au, Al, Ag NPs on Si/glass	Rhodamine 6G in PMMA	423	[138]

**Table 3:** Unit cell of metasurfaces, detected molecules, and the enhancement factor (EF) for SERS using dielectric and hybrid metasurfaces (DTNB = 5,5′-dithio-bis-[2-nitrobenzoic acid]; NPs = nanoparticles; CV = crystal violet).

Unit Cell of Metasurfaces	Detected Molecules	EF	Reference
Tip-shaped Si (micro-cones)	DTNB molecules and AuNPs	$10^{11}$	[139]
Square lattice nano-holes in Si_3_N_4_ on SiO_2_	CV in ethanol	$10^{3}$	[140]
Two tilted ellipses of TiO_2_	Methylene Blue	$10^{3}$	[141]
Al_2_O_3_/MgF_2_ metagrating	Raman-active analytes	$10^{7}$	[142]

**Table 4:** Comparison of plasmonic and dielectric metasurfaces for surface-enhanced spectroscopy.

Aspect	Plasmonic Metasurfaces	Dielectric Metasurfaces
**Main resonance mechanism**	localized surface plasmons (LSPs), surface plasmon-polaritons (SPPs)	Mie-type electric, magnetic, and toroidal multipole resonances
**Typical materials**	Au, Ag, Al, Cu	Si, Ge, TiO_2_
**Field enhancement**	Very high in nanogaps (hot-spots) owing to strong plasmonic near-fields	Moderate; directional and tunable through multipolar interference
**Optical losses**	High, especially in the visible and NIR, because of ohmic damping in metals	Low; high-index dielectrics provide reduced loss in VIS–NIR regions
**Spectral tunability**	Limited; constrained by material dispersion and damping	High; enabled by geometric design and modal engineering
**Chemical stability**	Lower; prone to oxidation (particularly Ag, Al) and surface degradation	Higher; oxides and semiconductors offer good chemical stability
**Biocompatibility and surface chemistry**	Au is biocompatible; Ag is cytotoxic and oxidises; strong Au–thiol interactions	Generally inert; allows silanisation; no galvanic toxicity
**Fabrication complexity**	High precision required for nanogaps and reproducible hot-spots	More fabrication-tolerant; periodic patterns often sufficient
**CMOS compatibility**	Limited; integration with silicon photonics is challenging	High; compatible with standard CMOS and on-chip photonic platforms
**Distance-dependent effects**	<5 nm optimal for SERS/SEF; strong quenching if molecule is too close	Effective up to 20–30 nm; no metal-induced fluorescence quenching
